# Exploring Defect-Engineered Metal–Organic Frameworks with 1,2,4-Triazolyl Isophthalate and Benzoate Linkers

**DOI:** 10.1021/acs.inorgchem.4c01589

**Published:** 2024-05-29

**Authors:** Sibo Chetry, Muhammad Fernadi Lukman, Volodymyr Bon, Rico Warias, Daniel Fuhrmann, Jens Möllmer, Detlev Belder, Chinnakonda S. Gopinath, Stefan Kaskel, Andreas Pöppl, Harald Krautscheid

**Affiliations:** † Faculty of Chemistry and Mineralogy, Universität Leipzig, Johannisallee 29, Leipzig 04103, Germany; ‡ Felix-Bloch-Institute of Solid-State Physics, Faculty of Physics and Earth Sciences, Universität Leipzig, Linnéstrasse 5, Leipzig 04103, Germany; § Faculty of Chemistry and Food Chemistry, Department of Inorganic Chemistry I, Technische Universität Dresden, Bergstrasse 66, Dresden 01069, Germany; ∥ Catalysis and Inorganic Chemistry Division, CSIR − National Chemical Laboratory, Dr Homi Bhabha Road, Pune 411 008, India; ⊥ Institut für Nichtklassische Chemie e.V., Permoserstraße 15, Leipzig 04318, Germany

## Abstract

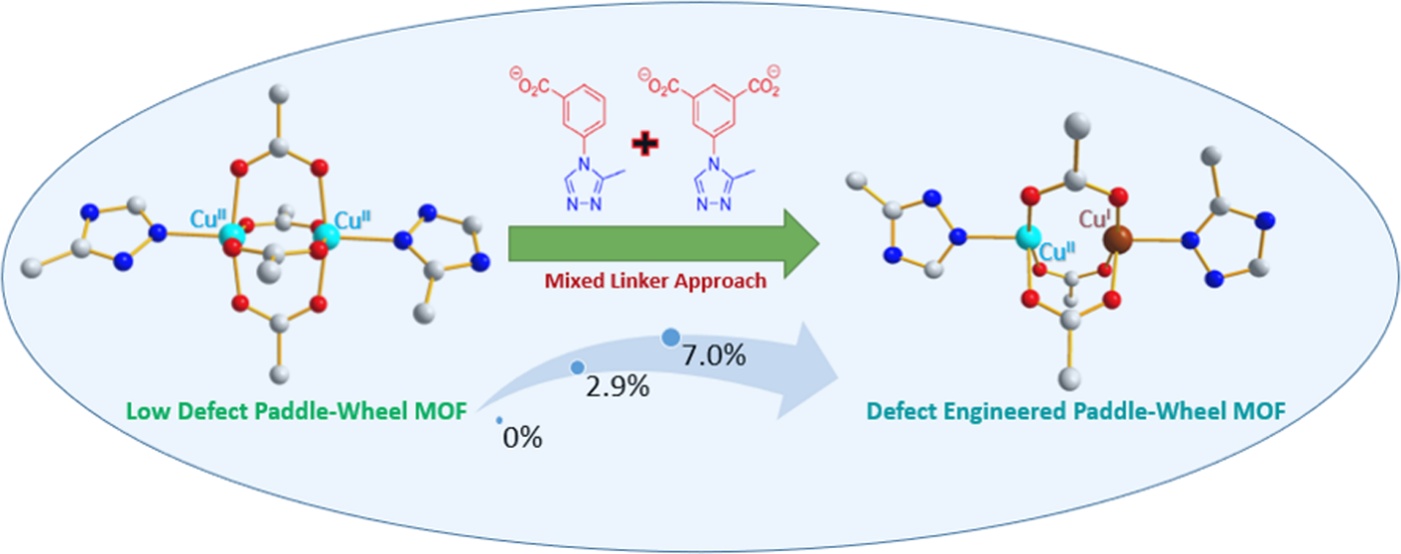

Synthesis and characterization of DEMOFs (defect-engineered metal–organic frameworks) with coordinatively unsaturated sites (CUSs) for gas adsorption, catalysis, and separation are reported. We use the mixed-linker approach to introduce defects in Cu_2_-paddle wheel units of MOFs [Cu_2_(Me-trz-ia)_2_] by replacing up to 7% of the 3-methyl-triazolyl isophthalate linker (^1^L^2–^) with the “defective linker” 3-methyl-triazolyl m-benzoate (^2^L^–^), causing uncoordinated equatorial sites. PXRD of DEMOFs shows broadened reflections; IR and Raman analysis demonstrates only marginal changes as compared to the regular MOF (ReMOF, without a defective linker). The concentration of the integrated defective linker in DEMOFs is determined by ^1^H NMR and HPLC, while PXRD patterns reveal that DEMOFs maintain phase purity and crystallinity. Combined XPS (X-ray photoelectron spectroscopy) and *cw* EPR (continuous wave electron paramagnetic resonance) spectroscopy analyses provide insights into the local structure of defective sites and charge balance, suggesting the presence of two types of defects. Notably, an increase in Cu^I^ concentration is observed with incorporation of defective linkers, correlating with the elevated isosteric heat of adsorption (Δ*H*_ads_). Overall, this approach offers valuable insights into the creation and evolution of CUSs within MOFs through the integration of defective linkers.

## Abstract 2

This work demonstrates that introducing defects into ultramicroporous MOFs via a mixed-linker approach markedly elevates their adsorption properties. This method, focusing on the strategic placement of CUSs within Cu_2_-paddle wheel units, leads to partial reduction of Cu^II^ to Cu^I^ and to an enhanced isosteric heat of H_2_ adsorption. The research underscores the significance of defect engineering in MOFs to modify MOF properties.

## Introduction

Metal–organic frameworks (MOFs) as one class of porous materials have attracted sustained interest over the past 25 years; their formation can be described as metal nodes (SBU) connected by organic linkers.^1^ These materials exhibit remarkable properties, including a high surface area, tunable porosity, and the potential for functionalization of coordination space, which make them superior candidates for gas storage, molecular separation,^2,3^ heterogeneous catalysis,^4^ sensing,^4^ etc. There is enormous interest arising in defect-engineered MOFs (DEMOFs).^5−13^ It is currently understood that defects can be used to alter the band gap,^14,15^ electrical conductivity,^16−18^ gas adsorption,^7,12−17^ and catalytic properties of MOFs.^19−21^ Experimental and theoretical studies have demonstrated that a controlled presence of structural defects can positively influence the physical and chemical characteristics of MOF materials.^22−25^ These defects can be a result of missing metal nodes or missing linkers, which can disrupt the regularity of the framework locally and result in the absence of either the full molecule or only a subset of functionalities when fragmented linkers are integrated.^9,26,27^ Defects can be generated in the MOFs by applying various strategies that are well established in the literature, i.e., de novo synthesis and postsynthetic^7^ treatment. The solid-solution approach in de novo synthesis allows for the integration of defects and modifications during the MOF’s formation, providing a uniform and precise distribution of components. This method has been used with success to produce DEMOFs.^23,28−32^ The nature of the incorporated linkers determines the type of resulting mixed-linker MOF, while the crystalline phase and topology of the framework are usually maintained.^33^ For example, the synthesis of HKUST-1 in the presence of pyridine dicarboxylic acid as a defective linker results in point defects and leads to the formation of Cu^I^ sites inside the framework due to charge compensation. Defect-HKUST-1 has been thoroughly characterized using *in situ* IR spectroscopy, X-ray photoelectron spectroscopy, and *cw* EPR spectroscopy.^12,13,34,35^ The importance of IR spectroscopy and Raman spectroscopy in characterizing DEMOFs is also demonstrated in the literature, which offer in-depth understanding of the types of defects and how they affect MOF characteristics.^12,13,34−36^

MOF linkers combining neutral 1,2,4-triazole and anionic carboxylate groups offer several benefits: low overall charge, resistance to oxidizing agents, rich coordination chemistry, and various possibilities for functionalization.^37−39^ Due to these characteristics, MOFs containing these ligands might be valuable in catalysis, gas adsorption, or separation of gases. For example, the coordination polymer [Cu(Me-4py-trz-ia)], (Me-4py-trz-ia^2–^ = 5-(3-methyl-5-(pyridin-4-yl)-4*H*-1,2,4-triazol-4-yl)isophthalate) is a representative for a family of MOFs for the separation of methane from nitrogen; this MOF also exhibits one of the highest hydrogen uptakes at atmospheric pressure (3.1 wt % at 77 K).^40,41^ [Cu_2_(^1^L)_2_]^42^ (^1^L^2–^ = 5-(3-methyl-4*H*-1,2,4-triazol-4-yl)isophthalate) belongs to third-generation MOFs with a characteristic ultramicropore system, and this MOF showcased a characteristic dynamic behavior, i.e., guest induced structural change of the host lattice.^42,43^ [Cu_2_(^1^L)_2_] is reported to adsorb CO_2_ up to 7.3 mmol/g at 298 K and 3 MPa, and in a prior investigation, it was determined that the pore size distribution (PSD), derived from the crystal structure, exhibits a bimodal pattern with pore sizes of 0.49 and 0.32 nm, whereas at 77 K, no N_2_ adsorption is observed after activation^42^; more detailed studies on *n*-butane sorption in this structurally flexible MOF have been reported recently.^43,44^

In the following, we report the synthesis of defect-engineered MOFs (DEMOFs) by mixing the regular linker ^1^L^2–^ with 3-(3-methyl-4*H*-1,2,4-triazol-4-yl)benzoate^37^ (^2^L^–^) as a “defective” linker. Due to the missing carboxylate group, incorporation of ^2^L^–^ should generate unoccupied coordination sites in the equatorial position of the Cu_2_(carboxylate)_4_ paddle wheel units and should also lead to the preferred formation of Cu^I^ sites inside the framework for charge compensation. More sensitive and suitable techniques are needed to gain a deeper knowledge of the local structure and electronic alterations at the defective sites. Such tools are X-ray photoelectron spectroscopy, electron paramagnetic resonance (EPR) spectroscopy, and X-ray absorption spectroscopy (XAS).^12,45–50,51^ For example, studies by Fang et al.,^12^ Marx et al.,^51^ and Kjaervik et al.,^52^ utilized XPS, FT-IR, and XAS measurements to demonstrate the influence of doping in HKUST-1 with the modified linker 2,5-pyridinedicarboxylate (PyDC) in defect-engineered HKUST-1. EPR methods have been shown to be powerful tools for studying the local structure of triazolyl benzoate-based MOFs.^45,46^ This class of linkers leads to the formation of dinuclear metal ion paddle wheel units that exhibit typical magnetic exchange interactions between Cu^II^ atoms; these are primarily explained by the indirect coupling through several ligand orbitals, known as the superexchange path.^46^ Since the past decades, *cw* EPR spectroscopy has proven to be a sophisticated tool to probe such magnetic interactions in dinuclear Cu^II^ units in MOFs and other types of magnetic materials.^45−50^ The determination of the isosteric heat of adsorption Δ*H*_ads_^57^ is an important method, notably in MOFs, to better verify the creation of coordinatively unsaturated sites^13^ (CUSs) within the MOF structure. The basic idea is that a larger concentration of these sites causes a rise in the amount of energy gained during adsorption or needed for desorption.^61^ These investigations demonstrate a relationship between Δ*H*_ads_ and the concentration of CUSs. This relationship has important ramifications since it allows us to quantitatively evaluate the distribution of coordinatively unsaturated sites within MOFs, providing important information about their structural characteristics and possible uses. Therefore, we explore in detail the magnetostructural properties by *cw* EPR, local sites by XPS, and CUSs by determination of Δ*H*_ads_. This research contributes to advancing the understanding and utilization of defect-engineered MOFs in various applications.

## Experimental Description

### Synthesis of the Defect-Engineered MOFs

**Scheme 1:**
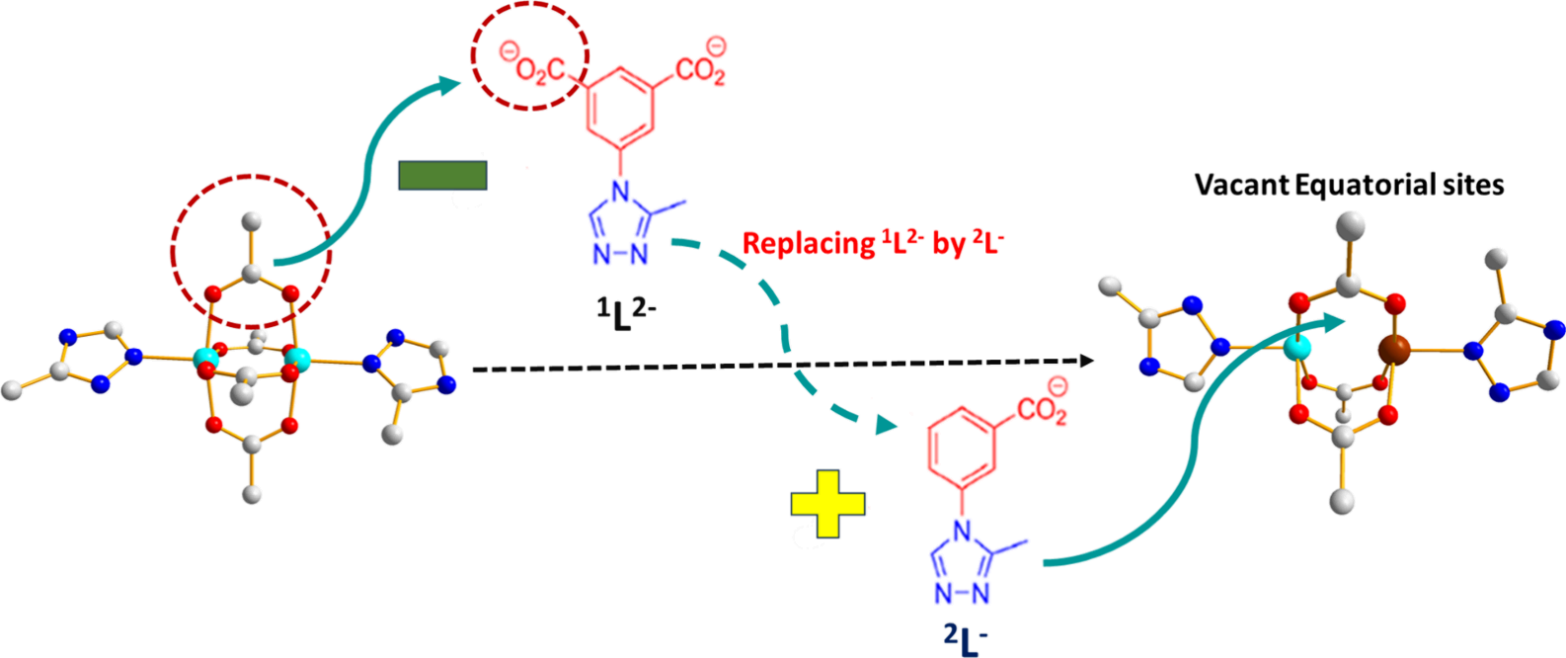
The Strategy Implemented for the Development of DEMOFs is Based on Partial Replacement of the Regular Linker ^1^L^2–^ by the “Defective Linker” ^2^L^–^ Lacking One Carboxylate Group The diagram focuses on Cu–Cu paddlewheel unit changes due to ^2^L^–^ incorporation. Color coding: Cu^I^, brown; Cu^II^, turquoise; C, gray; O, red; and N, blue. Additional structural specifics are excluded for the sake of simplicity.

Synthesis of the linkers H_2_(Me-trz-ia) (H_2_^1^L) and H(Me-trz-mba) (H^2^L) as well as the DEMOFs synthesis were performed according to Kobalz et al.^42^ Details are reported in the Supporting Information (SI, Sections S2 and Section S3). For synthesis of the DEMOFs we modified the procedure by replacing up to 24% (in the reaction mixture) of the regular linker H_2_^1^L by the defective linker H^2^L while keeping the molar ratio Cu^II^: ligand = 1:1 constant (Scheme 1 and SI, Table S1). After synthesis, the samples were thoroughly washed with DMF:EtOH (1:1) followed by solvent exchange for 7 days. During this time MeOH was replaced regularly every 6 h. With an increasing percentage of H^2^L, a slight color change of the resulting DEMOF from green to bluish-green is observed (SI, Figure S1). After solvent exchange, the samples were kept under MeOH inside a sealed vial for further analysis. Samples without any defective linker are referred to as ″**ReMOF**″ (regular MOF) while samples with a defective linker are named based on the actual percentage of defective linker incorporated. For instance, a sample [Cu_2_(^1^L_1–x_^2^L_*x*_)_2_] containing 2.9% defective linker (x = 0.029), obtained from HPLC analysis, is labeled as ″**DEMOF_2.9%**″.

## Results and Discussion

Solvothermal synthesis of [Cu_2_(^1^L_1–*x*_^2^L_*x*_)_2_] was achieved by using CuCl_2_·2H_2_O, H_2_^1^L, and H^2^L at different molar ratios while keeping the copper salt amount fixed.

### PXRD Analysis

**Figure 1:**
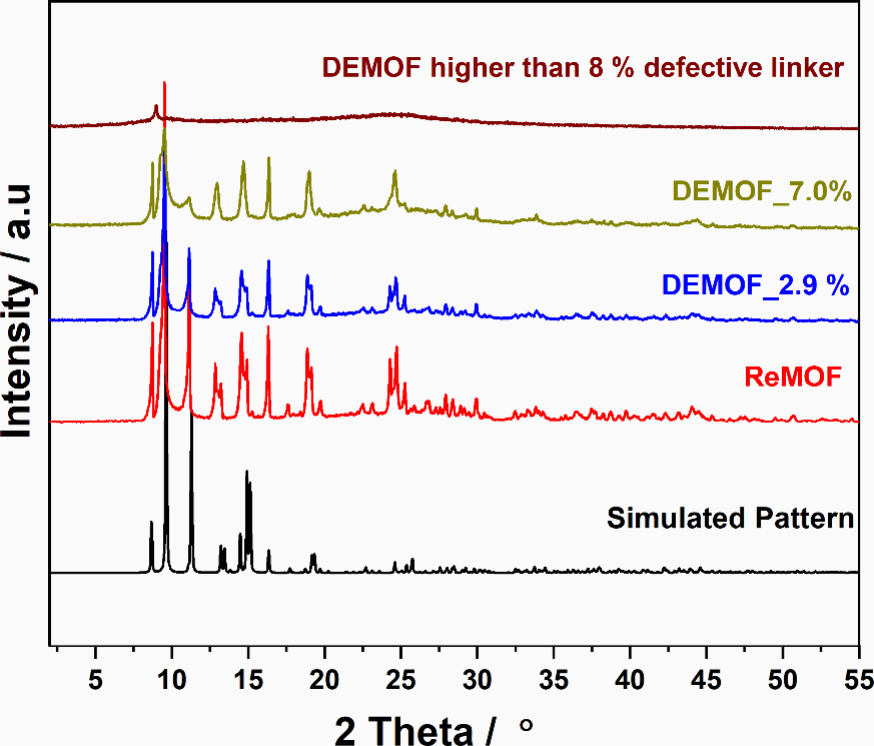
PXRD pattern (r.t., Cu–K_α1_ radiation) of solvent-exchanged ReMOF and DEMOF samples. The simulated pattern is based on single crystal data measured at 180 K.^42^

The powder XRD patterns of DEMOF_2.9% and DEMOF_7.0% are in good agreement with the simulated patterns originating from single crystal data of ReMOF [Cu_2_(^1^L)_2_],^42^ and no extra phases are detected (Figure 1). The diffraction patterns also show that the parent MOF structure is retained with incorporated ^2^L^–^ up to certain limits (less than 8%). With an increasing percentage of ^2^L^–^ in the DEMOF, we observe a decrease in intensity as well as broadening of the peaks; the peak around 2Θ = 9.2° becomes broader and several reflections disappear with a higher percentage of ^2^L^–^. This is expected due to the formation of defects based on structural irregularities inside the DEMOF. A concentration of ^2^L^–^ higher than 8% leads to the formation of an amorphous product, suggesting that the DEMOF system does not feature a mixture of MOF phases consisting of ReMOF and an additional compound containing the defective linker ^2^L^–^. Instead, up to the ^2^L^–^ concentration in DEMOF_7.0%, we observe a crystalline product through PXRD analysis, confirming that the DEMOF samples can incorporate ^2^L^–^ alongside the regular linker ^1^L^2–^.

The microstrain broadening^53^ in the PXRD patterns of DEMOFs shows a positive correlation with an increasing percentage of ^2^L^–^ in DEMOF samples; details are reported in the SI (Section S5.2, Figure S6). This empirical evidence supports the presence of internal defects in the DEMOF samples.

### FTIR and Raman Spectroscopy

**Figure 2:**
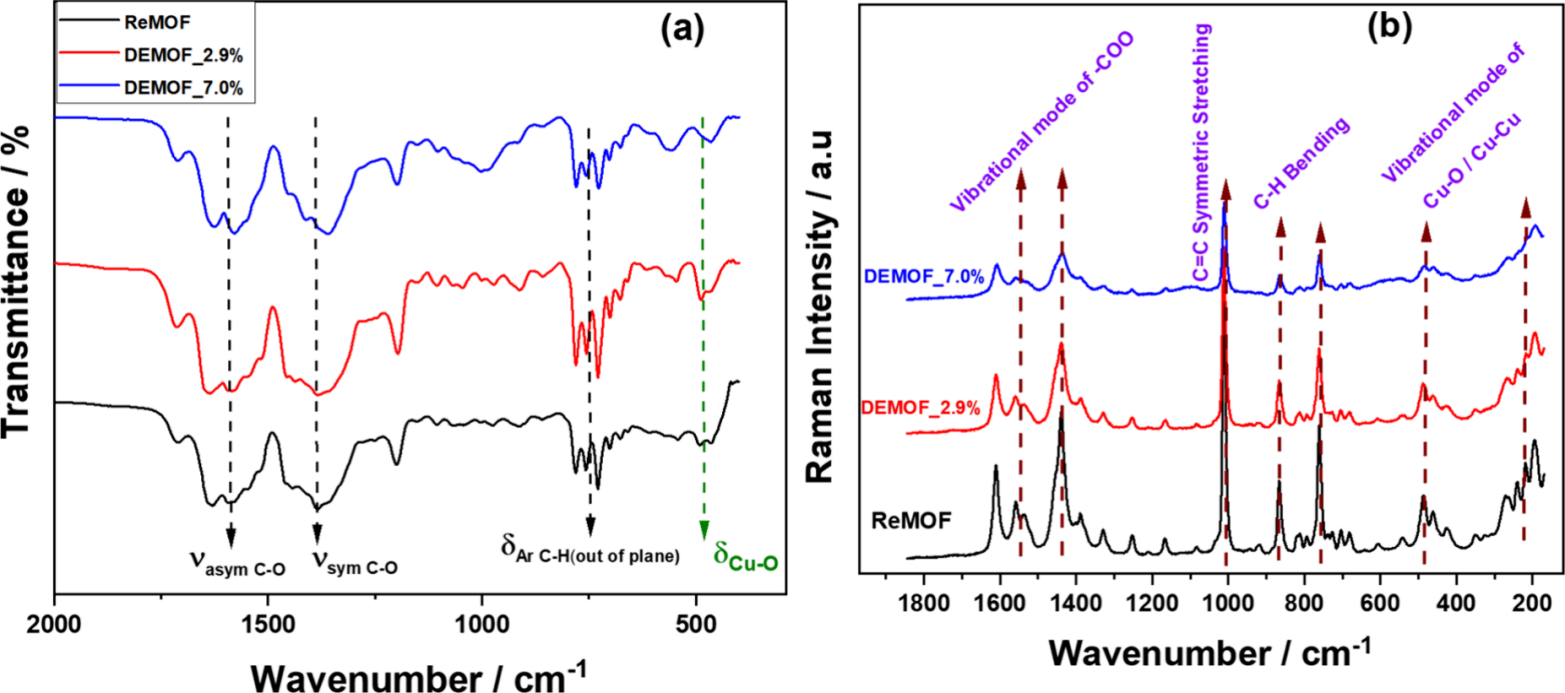
(a) FTIR spectra of ReMOF and DEMOF samples (δ: bending, ν_sym_: symmetric stretching, ν_asym_: antisymmetric stretching, τ: twisting) and (b) Raman spectra of ReMOF and DEMOF samples with vibrational frequency assignments.

FTIR and Raman spectra were obtained after sample activation (Figure 2a,b). Comparative analysis of the FTIR spectra between the ReMOF and DEMOF revealed minor differences attributed to the absence of a carboxylate group in the ^2^L^–^ linker. We observed a small red-shift in DEMOF_7.0% as compared to the ReMOF. The complete spectra with assignments are available in the SI (Section S6 and Figure S10). These findings suggest that the incorporation of ^2^L^–^ introduces defects, leading to the observed broadening as well as the lowering of wavenumbers in the IR spectra. A similar broadening and red-shift are also reported by Fang et al.^12^ in their work on defect-engineered HKUST-1 using a mixed linker approach.

The peak assignments in the Raman spectra are included in Figure 2b and the SI (Section S7). Similar to the IR spectra, there is a broadening of the Raman signals of the DEMOFs. We can assign the signal between 190 and 270 cm^**–**1^ to the Cu–Cu dimer stretching mode,^36^ which is an IR-inactive vibration mode. Again, the spectra of the ReMOF and DEMOFs demonstrate notable broadening and reduced intensity in the stretching vibration, accompanied by a slight red shift. These observed effects resemble the well-documented phenomena observed in various inorganic materials.^54−56^ Therefore, we attribute these changes to defects in the paddle wheel unit of the MOFs due to the incorporation of the defective linker ^2^L^–^.

### Determination of the Amount of Incorporated Defective Linker ^2^L^–^ in [Cu_2_(^1^L_1–*x*_^2^L_*x*_)_2_]

**Figure 3:**
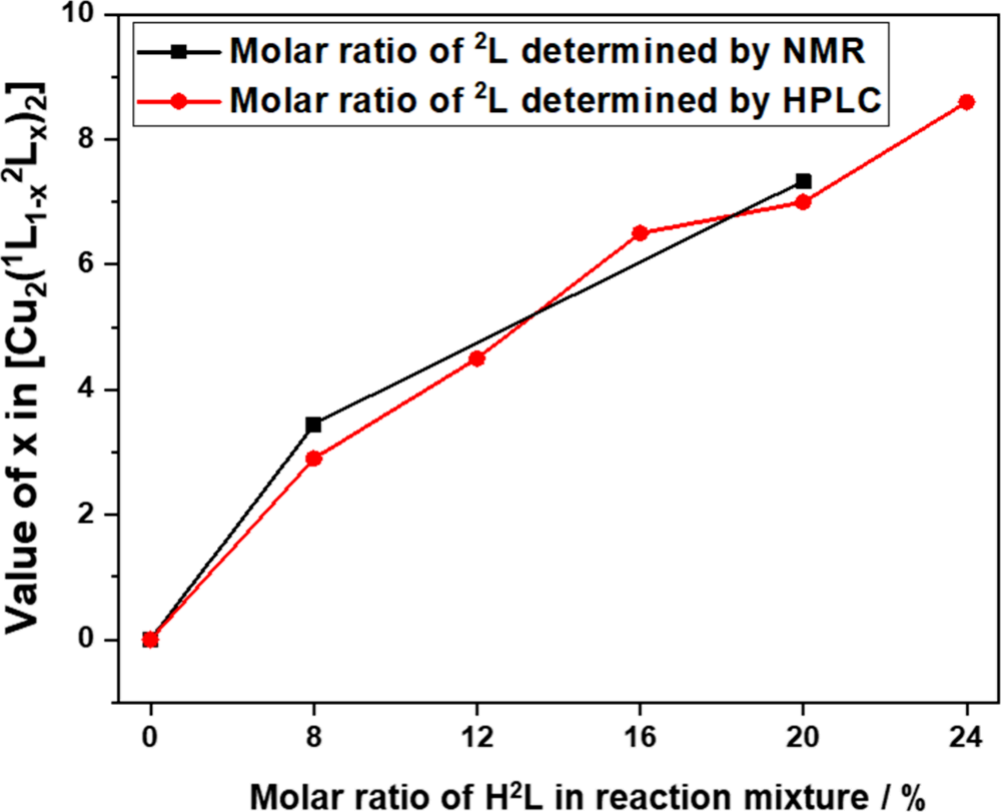
Molar ratio *x* = ^1^L^2–^/(^1^L^2–^ + ^2^L^–^) in the [Cu_2_(^1^L_(1–*x*)_^2^L_*x*_)_2_] DEMOF as determined by integration of ^1^H NMR signals (black) and by HPLC (red) vs H^2^L/(H_2_^1^L + H^2^L) in the reaction mixture for synthesis.

The amount of linker ^2^L^–^ incorporated in the DEMOFs [Cu_2_(^1^L_(1–*x*)_^2^L_*x*_)_2_] was determined by digestion of the synthesized MOFs in NaOH/D_2_O (SI, Section S8) and subsequent analysis by ^1^H NMR spectroscopy and HPLC (SI, Section S9). In the ^1^H NMR spectra of the DEMOF samples after digestion, slight differences in the chemical shift of protons of ^2^L^–^compared to the regular linker ^1^L^2–^ (SI, Section S8) are due to a different chemical environment. Slight changes in chemical shifts also occurred in comparison to the spectra of pure carboxylic acids H_2_^1^L and H^2^L after deprotonation in the basic digestion medium. The fraction of ^2^L^–^ within the DEMOF depends on the feeding ratio H^2^L/H_2_^1^L + H^2^L in the synthesis as documented in Figure 3 and the SI (Section S8, Table S2 and Section S9, Table S3).

The findings presented herein demonstrate the capacity to finely modulate the incorporation of ^2^L^–^ through deliberate adjustments in the feeding ratio. The quantity of ^2^L^–^ encompassed within the framework is lower than the initial amount introduced in the reaction mixture, and the molar ratio increases almost linearly with respect to the linker ratio during synthesis (Figure 3). Even though ^1^H NMR spectroscopy is a reliable method for discerning defective linkers, there are certain limitations such as overlapping peaks, changes in chemical shift, and the influence of possible remaining paramagnetic Cu^2+^ ions (SI, Section S8). As a complementary method of quantification, we employed high-performance liquid chromatography (HPLC) to precisely quantify the percentage of ^2^L^–^ incorporated into the DEMOF samples. More details about sample preparation and the mobile phase are provided in the SI (Section S9, Table S3). The quantifications based on ^1^H NMR and HPLC analysis are compared in Figure 3, and these results are in agreement within a deviation of ±0.5%. The percentage of ^2^L^–^ in the DEMOFs is included in their denominations as DEMOF_2.9% and DEMOF_7.0%.

### X-ray Photoelectron Spectroscopy (XPS)

**Figure 4:**
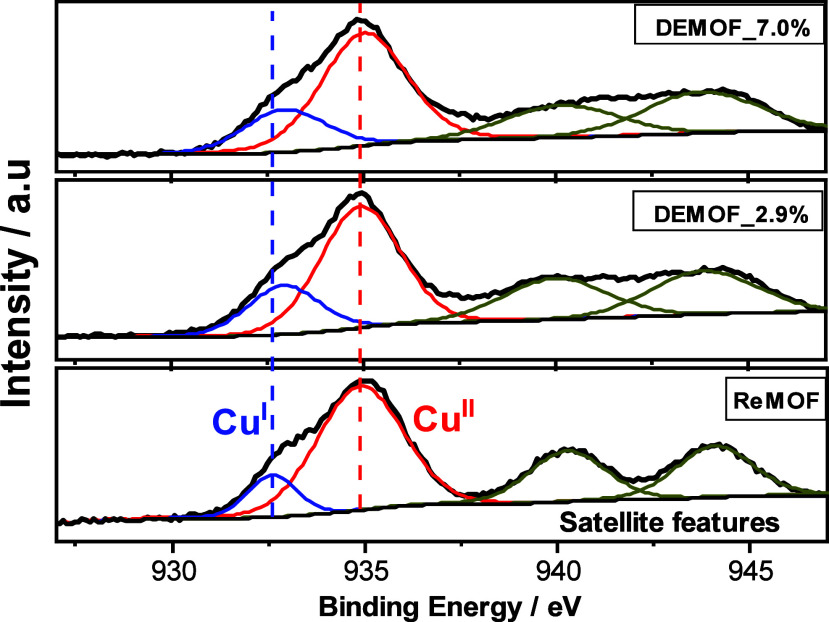
Cu 2p_3/2_ XPS spectra of the ReMOF and DEMOF samples. The line shape analysis and copper species quantification were performed using CASA XPS software.^62^

To gain quantitative information about the presence of Cu atoms in different oxidation states, Cu^I^ and Cu^II^, X-ray photoelectron spectroscopy was applied on ReMOF and DEMOF samples. Deconvoluted XPS spectra, binding energies, and the full assignments of the XPS spectra are described in detail in the SI (Section S11, Table S4). The Cu 2p_3/2_ peaks at 935.1 and 933.2 eV are attributed to Cu^II^ and Cu^I^, respectively (Figure 4). A Cu^I^ peak at 933.2 eV is already present in the ReMOF. Notably, Fang et al.^12^ assigned the peak around 933.2 eV to Cu^I^–Cu^II^ units formed due to Cu^II^ reduction to Cu^I^ in paddle wheel structures (defective paddle wheel unit) and the peak at 935.1 eV to regular Cu^II^–Cu^II^ paddle units without defects. The XPS spectra clearly show an increasing increment of the area within the shoulder peak of Cu^I^ from the ReMOF to DEMOF_7.0%, which implies increasing defect concentration as well as heterogeneity in the DEMOF due to defective linker incorporation. Our results of the estimated increment of Cu^I^ agree well with the analysis of DEMOFs, as reported in the literature.^12,13^ The presence of two different types of paddle wheel units is also supported by broad Cu LMM Auger spectra, which exhibit signals around 916.5 eV,^12,58−60^ assigned to defective paddle wheels Cu^I^–Cu^II^, and 918.2 eV for regular Cu^II^–Cu^II^ paddle wheels (SI, Section S11.1, Figures S16 and S17). The XPS spectra provide characteristic peaks corresponding to both Cu^I^ and Cu^II^ oxidation states. Although the relative ratios of these peaks can be used to quantify the abundance of Cu^I^ from defective paddle wheel units within the MOF, the quantifications may have some uncertainty due to X-ray-induced degradation of Cu^II^ species or due to the surface charge effect.^52^ Cu^I^ species might be formed already during synthesis or in the activation process where ethanol might serve as the reducing agent.^12,52^ As the incorporation of defective linkers ^2^L^–^ with a missing carboxylate group increases, the number of Cu^I^ defective paddle wheel units also increases. Consequently, the proportion of coordinatively unsaturated sites (CUSs) within the MOF structure is also assumed to increase. Peak broadening and small shifts in the positions of the peaks indicate the presence of different oxidation states or a change in coordination environments of the copper ions associated with the defect sites. We notice a drastic increase in the Cu^I^ population from the ReMOF to DEMOF_2.9%; however, the changes to DEMOF_7.0% are not significant (Figure 4). The relative abundance of defect sites, estimated by the relative intensities of the peaks associated with different oxidation states, is summarized in Table 1. These values might be biased by the surface sensitivity of XPS, which may not effectively reveal alterations occurring below the surface or within the bulk of the DEMOFs. To address the limitations of XPS in analyzing these subsurface changes, we utilized *cw* EPR, a potent characterization technique. This method enables a more thorough investigation of such defective sites, which are discussed in the subsequent section.

**Table 1:** XPS Analysis for the Relative Quantification of Cu^II^ and Cu^I^ in ReMOF and DEMOF Samples

**sample**	**oxidation state**	**percentage**
ReMOF	Cu^II^	83.5%
	Cu^I^	16.5%
DEMOF_2.9%	Cu^II^	73.4%
	Cu^I^	26.6%
DEMOF_7.0%	Cu^II^	73.8%
	Cu^I^	26.2%

### Electron Paramagnetic Resonance (EPR)

**Figure 5:**
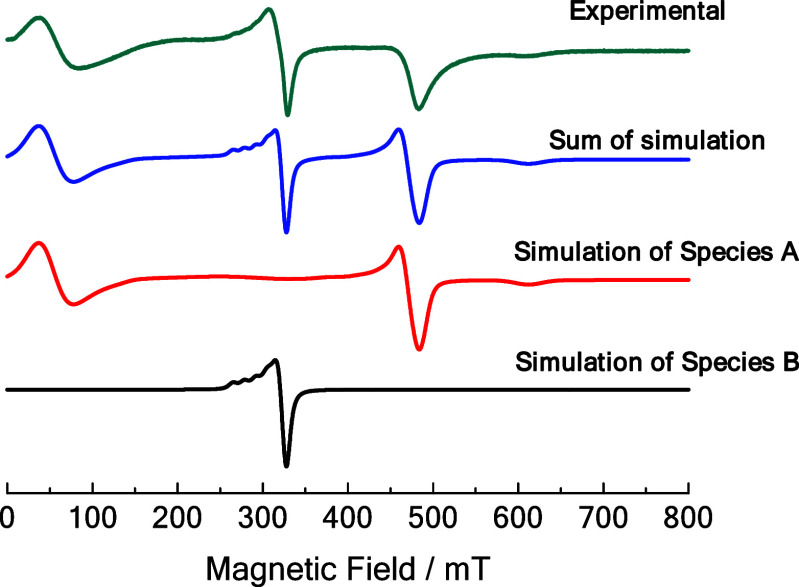
X-band *cw* EPR spectra of the ReMOF [Cu_2_(^1^L)_2_] measured at 160 K and its spectral simulation of [Cu_2_(^1^L)_2_] by considering contributions of two different species, species A with *S* = 1 and species B with *S* = 1/2.

The X-band *cw* EPR spectra of the ReMOF [Cu_2_(^1^L)_2_] were measured by using powder samples suspended in methanol. The spectrum recorded at 160 K is provided in Figure 5, which consists of a superposition of the Cu^II^–Cu^II^ paddle wheel signal (species A) and the signal of uncoupled Cu^II^ species (B). Species A can be interpreted as two *S* = 1/2 spins from the interconnected Cu^II^–Cu^II^ dinuclear unit, resulting in antiferromagnetic coupling with *S* = 0 as the ground state level (diamagnetic) and *S* = 1 as the paramagnetic excited state level (triplet state). This triplet state (*S* = 1) is thermally populated above 60 K and gets more pronounced at higher temperatures, as observed in the temperature-dependent spectra obtained using *cw* X-band and Q-band EPR (SI, Section S12, Figure S18). The spectral simulation for the anisotropic *S* = 1 system can be described by the following Hamiltonian 1):
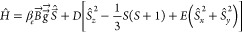
1where β_e_ denotes Bohr’s magneton and  denotes the Cu^II^–Cu^II^ pair *g*-tensor. *B*_0_ is the external magnetic field. Both *D* (axial) and *E* (orthorhombic) represent zero field splitting (zfs) parameters, where the zfs tensor and  tensor are assumed to be coaxial.  is the electron spin operator with *S* = 1. The axially symmetric system (*D* ≠ 0 and *E* = 0) has four expected transitions: *B*_x1,y1_, *B*_x2,y2_, *B*_z1_, and *B*_z2_, which can be clearly resolved at the Q-band (Figure S19). In the X-band frequency (9.4 GHz) at low magnetic fields (<150 mT), the *B*_x1, y1_ and *B*_z1_ transitions are superimposed with the signal of a forbidden transition (Δ*m*_s_ = ±2 with the magnetic spin quantum number *m*_s_) owing to the comparable magnitude of *D* and the microwave quantum frequency (Figure 5).

On the other hand, Q-band *cw* EPR provides a relatively more resolved spectral pattern as compared to the X-band since *B*_x1, y1_ and *B*_z1_ are separated from the typical forbidden transition (Δ*m*_s_ = ±2) signal around 500 mT (SI, Section S12, Figure S19). The fine structure pattern can be simulated using *g_xx,yy_* = 2.07, *g_zz_* = 2.38, *D* = 0.367 cm^–1^, and *E* ≈ 0 if we consider that *g*- and *D*- tensors are collinear. Those parameters are in good agreement with other compounds containing Cu_2_ paddle wheel units.^46,50^ The *D* parameters for the ReMOF and DEMOFs (Table 2) are also quite similar to each other, also implying comparable structures of the Cu_2_ paddle wheels.

**Table 2:** Comparison of Defect Ratio and Zero Field Splitting Parameters for ReMOF and DEMOF Samples, as Determined from the EPR Spectra

species	*N*_M_/*N*_PW_	*J* (cm^**–**1^)	*D* (cm^**–**1^)	*D* strain (cm^**–**1^)
ReMOF	0.12(2)	–252(30)	0.37(1)	0.030(5)
DEMOF_2.9%	0.29(2)	–200(30)	0.37(1)	0.030(5)
DEMOF_7.0%	0.33(2)	–220(30)	0.36(1)	0.040(5)

Temperature-dependent X-band and Q-band *cw* EPR spectra can only be applicable up to the freezing point of methanol at 175 K since beyond that temperature, the measurement is affected by a low S/N ratio. The *cw* EPR temperature-dependent data set is very useful for estimating the isotropic exchange coupling constant (*J*) of the antiferromagnetically coupled Cu^II^–Cu^II^ pairs using the Bleaney–Bowers equation (eq 2).^63^
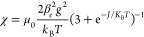
2where μ_0_ = 4π·10^–7^ T·m·A^–1^ is the permeability of vacuum, *g* is an average of the principle values of the *g*-tensor, β_e_ is the Bohr magneton, and *J* is the isotropic exchange coupling. One can consider that the magnetic susceptibility (χ) is proportional to the intensity of the *S* = 1 spectrum. As the line width of the *B*_x2,y2_ transition is temperature-independent, we used its signal amplitude as a measure for the relative EPR intensity, *I*_EPR_, of the *S* = 1 paddle wheel signal. The exchange coupling *J* is obtained by fitting the experimental temperature-dependent *I*_EPR_ data with eq 2. The estimated *J* values for the ReMOF and DEMOF do not differ significantly, ranging from −250 cm^**–**1^ for the ReMOF to −200 and −220 cm^**–**1^ for DEMOF_2.9% and DEMOF_7.0%, respectively (Figure S18). The negative sign of *J* indicates that the antiferromagnetic interaction of the Cu_2_ paddle wheels is still preserved in DEMOF samples.

**Figure 6:**
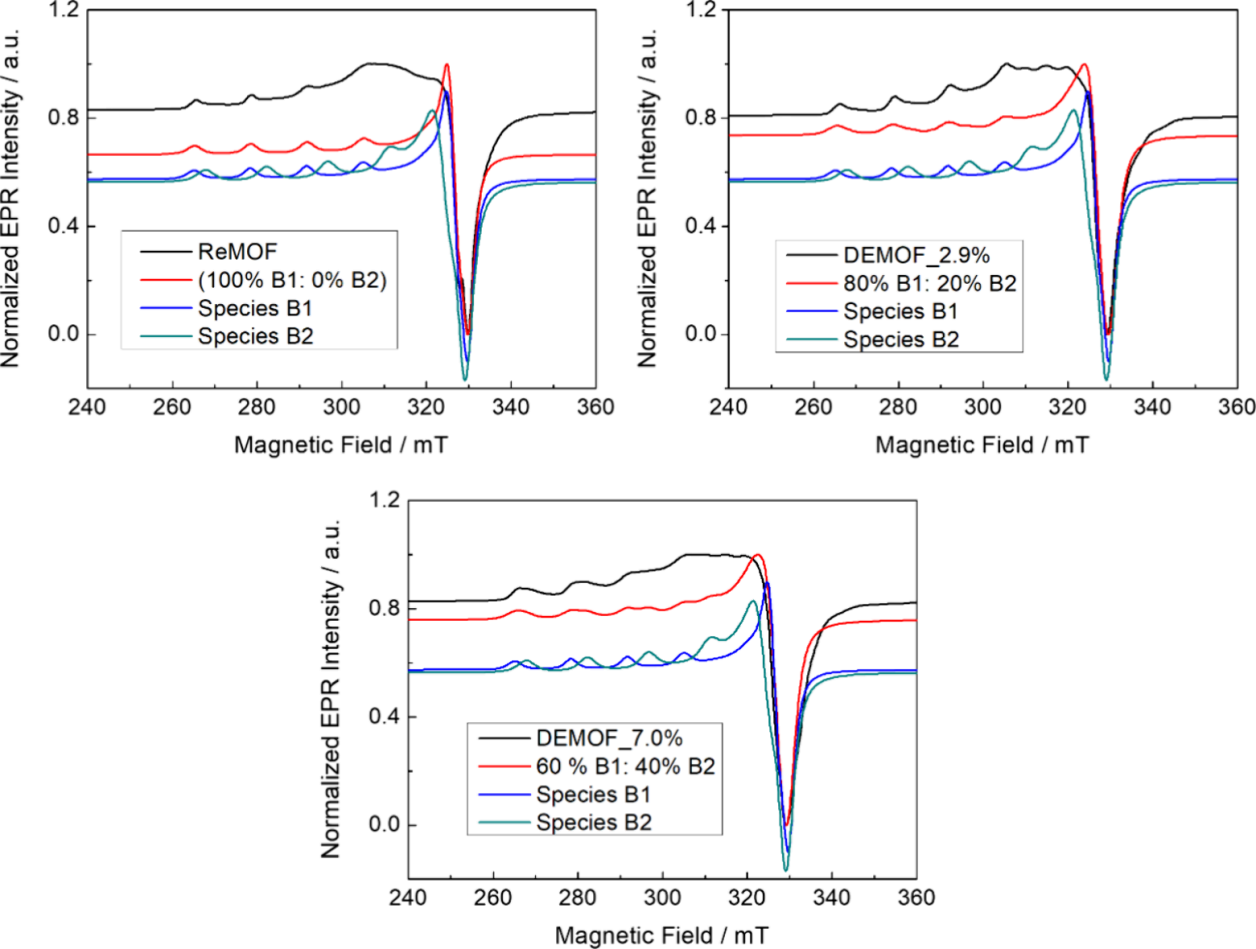
X-band *cw* EPR spectra of the *S* = 1/2 section (uncoupled Cu^II^ species) of the [Cu_2_(^1^L_1–*x*_^2^L_*x*_)_2_] MOF series measured at 10 K. The simulated spectrum in red is the total contribution of B1 and B2 species with the corresponding weight provided in the small insets.

The uncoupled Cu^II^ species B shows the typical anisotropic EPR powder pattern of an electron spin *S* = 1/2 coupled to a nuclear spin of Cu (*I* = 3/2) showing up in low-temperature spectra recorded at 10 K as four resolved hyperfine lines at *g_zz_* (*B*_0_ = 270–300 mT) (Figure 6). A complete set of *g* and *A* values obtained by Easyspin^64^ simulation is given in Table 3. As displayed in Figure 6, two uncoupled Cu^II^ species B1 and B2 with slightly different parameters are observed at 10 K. Species B1 can be used to describe the uncoupled Cu^II^ for the ReMOF sample, exhibiting a *g_zz_* value of 2.36, while the hyperfine coupling *A*_*zz*_ is accounted for 430 MHz. We can see a clear trend of the B2 species increasing as the amount of defective linker also increases from the spectral simulation results. The B2 species has slightly different EPR parameters: a *g_zz_* of 2.32 and *A*_*zz*_ of 460 MHz. For both B1 and B2, the spectral parameters are similar to those in literature reports on Cu^II^ compounds with a square pyramidal oxygen environment; the differences might be due to a slightly different charge delocalization.^48−50^

**Table 3:** Spin Hamiltonian Parameters of Mononuclear Cu^2+^ Ions in [Cu_2_(^1^L)_2_] ReMOF Samples Determined by Spectral Simulations of the Experimental EPR Spectra at 10 K Using Easyspin Software^64^

species	*g_xx,yy_*	*g_zz_*	*A*_*xx,y*y_ (MHz)	*A_zz_* (MHz)
B1	2.06(5)	2.36(4)	50(20)	430(20)
B2	2.06(5)	2.32(4)	50(20)	460(20)

**Figure 7:**
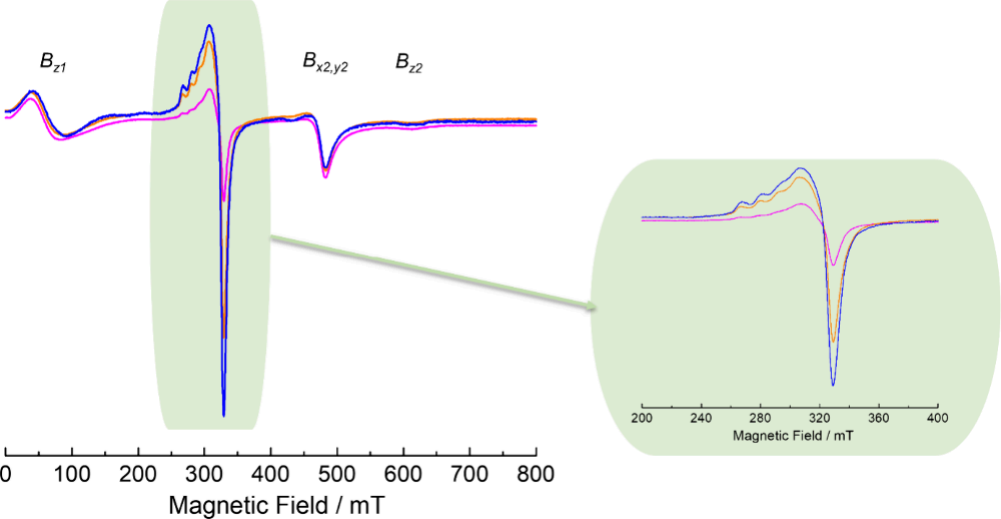
EPR spectra of the ReMOF (pink) in comparison to DEMOF_2.9% (orange) and DEMOF_7.0% (blue) recorded at X-band frequency and *T* = 160 K. All spectra were normalized to the intensity of species A for better comparison. The inset shows the comparison of signal intensities of the noncoupled Cu^II^ (B1, B2) from the abovementioned samples.

To verify the effect of defective linker introduction toward the properties of the MOFs, Figure 7 provides the spectral comparison of DEMOF samples with different amounts of defective linker ^2^L^–^. The spectral pattern at the X-band (Figure 7) changes significantly when the defective linker is incorporated. Obviously, the relative intensity of the EPR signals of species B1 and B2 with respect to signal A of the antiferromagnetically coupled Cu^II^–Cu^II^ paddle wheels increases with the increasing amount of defective linker. Taking further into account the square pyramidal coordination geometry of the Cu^II^ ion species belonging to signal B1, it is justified to assign these signals to a paramagnetic Cu^II^–Cu^I^ paddle wheel unit, which is a result of partial reduction of Cu^II^–Cu^II^ to Cu^II^–Cu^I^ due to ^2^L^–^ incorporation. In other words, species B2 can be regarded as coordinatively unsaturated sites (CUSs).

Assuming that the EPR signal intensity is proportional to the magnetic susceptibility χ and negligible differences are expected between *g*-values for both Cu species within the Cu_2_ paddle wheels, the intensity ratio of the signals of the noncoupled Cu^II^ species *I*_*M*_ and that of the paddle wheel unit *I*_*PW*_ can be estimated by fitting the weight (contribution) for each individual species, i.e., noncoupled Cu^II^ species (*I*_*M*_) and Cu_2_ paddle wheels (*I*_*PW*_), to the overall spectral simulation of the experimental spectra at 160 K. The spectral simulation for the ReMOF is given in Figure 5, whereas those for DEMOF_2.9% and DEMOF_7.0% are presented in the SI (Section S12, Figure S20). Thus, the ratio *N*_*M*_/*N*_*PW*_ can be extracted using eq 3.^50^ Note that *J* is an isotropic exchange coupling constant taken from the temperature-dependent data provided in the SI (Section S12, Figure S18).
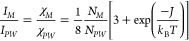
3

The ratio *N*_*M*_/*N*_*PW*_ is interpreted as the number of mononuclear Cu^II^ units with respect to Cu_2_ paddle wheel units. For the ReMOF, we found a ratio *N*_*M*_/*N*_*PW*_ around 0.12 (Table 2). This value is similar to those of some other Cu_2_ paddle wheel-based MOFs.^46,47,50^

Moreover, the ratio of defects is strikingly increased to 0.29 for DEMOF_2.9% (Figure 7). This implies that even a low percentage of defective linkers would impart a significant amount of defective sites. However, the ratio *N*_*M*_/*N*_*PW*_ for DEMOF_7.0% only enhanced slightly to 0.33 if compared to DEMOF_2.9%.

In general, we conclude that the trend of *N*_*M*_/*N*_*PW*_ ratio supports our assumption that the origin of the defect sites most probably stems from the structural defect, Cu^I^–Cu^II^ (defined as species B2 in Figure 6), which is promoted as a consequence of the charge compensation, which takes place with the incorporation of the defective linker ^2^L^–^ with one missing carboxylate group. These *N*_*M*_/*N*_*PW*_ ratios correlate well with the defect ratio determined by XPS analysis, taking into account the limitations because of surface sensitivity, and with the microstrain broadening observed by PXRD (SI, Section 5.2, Figure S6).

### H_2_ Gas Adsorption and Isosteric Heat of Adsorption

**Figure 8:**
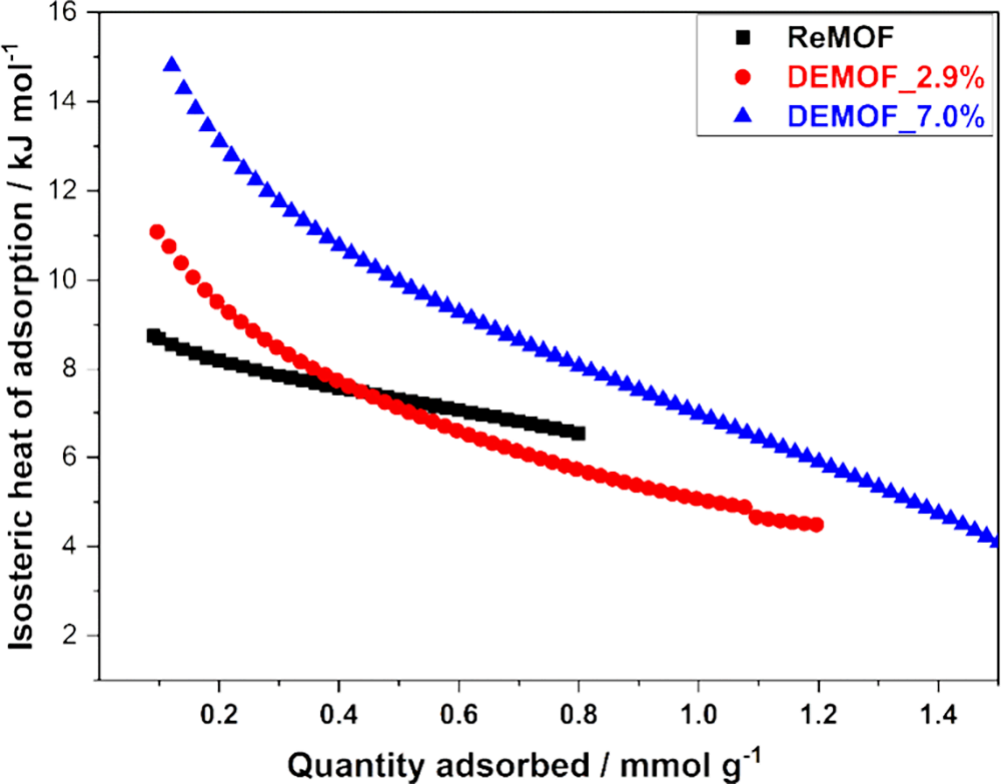
Δ*H*_ads_ obtained using Freundlich–Langmuir fits and Clausius–Clapeyron equation to the H_2_ desorption isotherms on ReMOF and DEMOF samples at 67, 77, and 87 K.

Understanding the strength of the interaction between adsorbate–absorbent (MOF) pairs requires knowledge of the isosteric heat of adsorption Δ*H*_ads_.^57^ It is also known that defects in MOFs as modified sites may alter the adsorption behavior.^9,65^ For the determination of Δ*H*_ads_ for H_2_ adsorption on both regular and defect-engineered materials, we conducted hydrogen physisorption experiments at three different temperatures, 67, 77, and 87 K. The observed hysteresis between adsorption and desorption branches of the isotherm could be interpreted as a sign of kinetically hindered adsorption in the ultramicropores.^66^ This is clearly reflected in the pressure profile (SI, Section S13, Figure S21) recorded during the adsorption and desorption branches. A long duration is needed for the equilibration in the adsorption process in the magnitude of 1 h for DEMOF_7.0% due to kinetic hindrance (SI, Figure S21). The slow adsorption kinetics at low temperatures originates from the narrow pore diameters and is more noticeable in ultramicroporous MOFs^66^ as a result of a kinetic convergency, molecular transport, and pore structure limitations. In contrast, the desorption process proceeds significantly faster. Therefore, similar to Weinrauch et al.,^10^ we used the desorption branch for the calculation of the isosteric heat of adsorption for hydrogen in the temperature range 67–87 K; see the SI (Section S13, Figure S23–S25) to understand the effects of defects. For the ReMOF, characterized by the lowest defect concentration, the desorption enthalpy spans from 8.7 kJ/mol (at a low uptake of 0.1 mmol/g) to 6.4 kJ/mol. On the contrary, DEMOF_2.9% exhibits a desorption enthalpy ranging from 11.1 to 4.5 kJ/mol. This rise in Δ*H*_ads_ at a low loading is attributed to CUS and Cu^I^ site formation through ^2^L^–^ incorporation. Compared to both the ReMOF and DEMOF_2.9%, DEMOF_7.0%, which features a higher concentration of the monoanionic linker ^2^L^–^, demonstrates even higher energies ranging from 14.8 kJ/mol at a low uptake to 4.0 kJ/mol at the higher uptake limit. This significant difference between the ReMOF and DEMOF_7.0% in the Δ*H*_ads_ parameter at the lower uptake range and the decrease with increasing H_2_ loading are shown in Figure 8. It can be interpreted that the adsorption of H_2_ takes place preferably on strong adsorption sites, which have been seen in many MOFs with coordinatively unsaturated open metal sites, and the subsequent adsorption occurs predominantly within the pores with lower adsorption/desorption enthalpy.^67^ According to this result, increasing the concentration of ^2^L^–^ leads to a higher concentration of structural defects, and these sites have stronger interactions toward the adsorbate. Various MOFs exhibit similar effects: Thomas et al.^68^ observed an enhanced interaction between H_2_ and Cu centers in an ultramicroporous MOF, leading to a high Δ*H*_ads_ for H_2_ adsorption of 12.3 kJ/mol. Likewise, synthesized derivatives of M-MOF-74 (M = Ni, Co, Fe, Mn, and Mg) with enhanced charge density in open metal sites, result in higher H_2_ binding enthalpies than M-MOF-74.^61^ For instance, Ni_2_(m-dobdc) and Co_2_(m-dobdc) had 12.3 and 11.5 kJ/mol, respectively, surpassing Ni_2_(dobdc) (11.9 kJ/mol) and Co_2_(dobdc) (10.8 kJ/mol).^61^ Notably, some cases with extraordinarily high Δ*H*_ads_ are reported, e.g., for Cu^I^-MFU-4l (32 kJ/mol) and V_2_Cl_2.8_(btdd) (21 kJ/mol).^10,61^ Therefore, the positive correlation can be directly evidenced by the introduction of CUSs, which is also supported by XPS and EPR studies in this work. These structural alterations, mainly stemming from defects, enhance the interactions between adsorbate molecules and the DEMOF.

In contrast, H_2_ adsorption experiments at 24 K showed that DEMOF_2.9% and DEMOF_7.0% adsorb significantly less hydrogen compared to their performance at 77 K. This loss of adsorption capacity could be attributed to reduced vibrations of the pore apertures at those low temperatures. With a more rigid aperture in the size of the H_2_ molecule, the guest molecules cannot enter the pores, as detailed in the SI (Section S13, Figure S29). This phenomenon has been observed in microporous MOF Mn(HCOO)_2_^69^ and particularly the findings by Oh et al.^70^ on Py@COF-1. COF-1 with incorporated pyridine shows a similar phenomenon. This behavior was explained as a unique temperature-activated gating mechanism that operates independent of structural alterations within the material’s framework. Instead, the gating effect is attributed to either a dynamic enlargement of the pore openings or the acquisition of sufficient kinetic energy by adsorbed molecules. This observation suggests that access to the pore is a direct consequence of thermal energy influencing the material’s behavior or the mobility of the molecules within.^69^ At higher temperatures, the vibrations allow passage of H_2_ molecules; thus, the gating behavior is controlled by the temperature through lattice vibrations, in contrast to gate-opening/closing behavior as observed, e.g., in MIL-53-type MOFs. If we compare our studies with Py@COF-1, the ReMOF, with less structural defects, permits hydrogen access at 24 K, showing a similarity to the inherently open structure of COF-1. On the other hand, DEMOF_7.0%, with its higher concentration of defects, mirrors Py@COF-1 behavior by selectively blocking hydrogen access at 24 K, a feature attributed to its more compact structure. Hence incorporation of ^2^L^–^ in the ReMOF, like the strategic inclusion of pyridine in parent COF-1, alters the MOF internal structure, leading to hindered gas diffusion. This parallel suggests that the presence and nature of defective linkers within MOFs can drastically influence gas adsorption by modifying the physical structure and affecting diffusion kinetics.

## Conclusions

In conclusion, the comprehensive analysis of DEMOF samples through XRD, EPR, XPS, and hydrogen adsorption, including determination of the isosteric heat of adsorption Δ*H*_ads_, elucidates the influence of defective linker incorporation in the MOF structure of [Cu_2_(^1^L)_2_] (^1^L^2–^ = 5-(3-methyl-4*H*-1,2,4-triazol-4-yl)isophthalate). The amount of incorporated linker ^2^L^–^ of 2.9% and 7.0% in DEMOFs [Cu_2_(^1^L_1–*x*_^2^L_*x*_)_2_] was quantitatively determined by ^1^H NMR spectroscopy and HPLC, showing the progressive increase in defective linker (^2^L^–^) concentration with increasing percentage of H^2^L during synthesis. The powder XRD patterns of DEMOF samples align well with the simulated pattern; with an increasing concentration of ^2^L^–^, intensity reduction and peak broadening are observed. FTIR and Raman analysis also support the qualitative aspects of defects with characteristic peak broadening with an increasing amount of ^2^L^–^. These are attributed to the formation of defects and irregularities within the DEMOFs, in agreement with increasing microstrain broadening detected in the PXRD patterns. A collapse of the crystalline framework is observed once the ^2^L^–^ concentration exceeds a certain threshold, leading to the formation of amorphous products.

According to XPS and EPR studies, the incorporation of the “defective linker” ^2^L^–^ with a missing carboxylate group in respect to the “regular linker” ^1^L^2–^ induces coordinatively unsaturated sites (CUSs). XPS analysis reveals the emergence of Cu^I^–Cu^II^ defective paddle wheel units, identified by distinct peaks in the spectra. While a consistent proportion of Cu^I^ is observed from the ReMOF to DEMOF_2.9%, due to surface sensitivity, XPS analysis is unable to detect significant changes in the defect structures at higher concentrations, such as in DEMOF_7.0%. In contrast, *cw* EPR X-band studies delve deeper into the defect dynamics, highlighting two specific species of Cu^II^ defects, termed B1 and B2. The intensity and prevalence of species B2, which represents coordinatively unsaturated sites resulting from a partial reduction from Cu^II^–Cu^II^ to Cu^II^–Cu^I^, increase with the addition of defective linkers. Therefore, while XPS effectively confirms the presence of Cu^I^ and Cu^II^, EPR completes these studies by offering detailed insights into the bulk structural changes associated with these defects. The investigation of isosteric heat of adsorption provides a quantitative understanding of defect-driven changes, and the CO_2_ adsorption reveals enhanced porosity in the DEMOF systems. Eventually, the incorporation of defective linkers and CUSs results in enhanced interactions between adsorbate molecules and DEMOFs.

Thus, the collective evidence suggests that the mixed linker approach leads to the formation of defects, which in turn enhance adsorption interactions. These findings provide valuable insights for tailoring the MOF properties through controlled defect engineering.

## Supporting Information Available

Analytical, spectroscopic, and other characterization data including chemicals, ligand synthesis, defect-engineered MOF synthesis, single crystal information, PXRD patterns and Pawley refinement, strain analysis, TGA, FTIR and Raman spectra,^1^H NMR, HPLC, XPS, *cw* EPR, H_2_ and CO_2_ gas sorption isotherms, isosteric heat of adsorption, SEM images, and elemental analysis data (PDF)
