# Supplementary material for: Exploring Defect-Engineered Metal–Organic Frameworks with 1,2,4-Triazolyl Isophthalate and Benzoate Linkers
Source: Inorg Chem. 2024 May 29;63(23):10843–53. doi: 10.1021/acs.inorgchem.4c01589 (PMC11167641; doi:10.1021/acs.inorgchem.4c01589)
Supplement: Supplementary file 1 — ic4c01589_si_001.pdf [file ic4c01589_si_001.pdf]

Exploring Defect Engineered MOFs with 1,2,4-Triazolyl Isophthalate and Benzoate Linkers

Sibo Chetry[a], Muhammad Fernadi Lukman[b], Volodymyr Bon[c], Rico Warias[a], Daniel Fuhrmann[a], Jens Möllmer[e], Detlev Belder[a], Chinnakonda S. Gopinath[d], Stefan Kaskel[c], Andreas Pöppl[b], Harald Krautscheid[a]*

[a] *Faculty of Chemistry and Mineralogy, Universität Leipzig, Johannisallee 29, 04103 Leipzig, Germany*

[b] *Felix-Bloch-Institute of Solid-State Physics, Faculty of Physics and Earth Sciences, Universität Leipzig, Linnéstrasse 5, 04103 Leipzig, Germany*

[c] *Technische Universität Dresden, Faculty of Chemistry and Food Chemistry, Department of Inorganic Chemistry I, Bergstrasse 66, 01069 Dresden, Germany*

[d] *Catalysis and Inorganic Chemistry Division, CSIR – National Chemical Laboratory, Dr Homi Bhabha Road, Pune 411 008, India,* and *Academy of Scientific and Innovative Research (AcSIR), Ghaziabad 201 002, India.*

[e] *Institut für Nichtklassische Chemie e.V., Permoserstraße 15, 04318 Leipzig, Germany*

**E-Mail: krautscheid@rz.uni-leipzig.de*

Table of Contents

S1. Chemicals 3

S2. Ligand Synthesis 4

S.2.1 Synthesis of Acetohydrazide 4

S2.2 Synthesis of 2-Methyl-1,3,4-oxadiazole 4

S2.3 Synthesis of 5-(3-Methyl-4H-1,2,4-triazole-4-yl) isophthalic acid ($H_2{}^1L$) 5

S2.4 Synthesis of 3-(3-Methyl-4*H*-1,2,4-triazole-4-yl) benzoic acid (H^2L) 5

S3. Defect Engineered MOFs (DEMOF) Synthesis 7

S4. Single crystal structure of ReMOF $[Cu_2({}^1L)_2]$ 8

S5. Powder X-ray Diffraction 9

S5.1 Powder X-ray diffraction (XRD) of ReMOF and DEMOFs 9

S5.2 Strain effect 11

S5.3 Pawley Refinement 12

S6. FTIR Spectroscopy 14

S7. Raman Spectroscopy 15

S8. NMR Spectroscopy 16

S9. HPLC Analysis 19

S10. Simultaneous Thermal Analysis (TG-MS) 20

S11. X-ray Photoelectron Spectroscopy (XPS) 22

S12. Electron Paramagnetic Resonance (EPR) 24

S13. Gas Adsorption, Isosteric Heat of adsorption 28

S14. Scanning Electron Microscopy (SEM) Images 37

S15. Elemental Analysis 40

S16. References 45

S1. Chemicals

All chemicals were commercially available.

Chemical	Formula	Vendor	Purity
Ethyl acetate (EtOAc)	$C_4H_8O_2$	VWR HiPerSolv	99.99%
Hydrazine monohydrate	$N_2H_4 \cdot H_2O$	Acros Organics	100 % (64 % Hydrazine)
Ethanol (EtOH)	C_2H_6O	VWR HPLC Grade	99.99%
para-Toluenesulfonic acid (*p*TsOH)	$C_7H_8O_3S$	Fluka Analytical	98%
5-Amino-1,3-isophthalic acid	$C_8H_7NO_4$	Acros Organics	99.95%
3-Aminobenzoic acid	$C_7H_7NO_2$	Acros Organics	99.95%
Copper(II)-chloride dihydrate	$CuCl_2 \cdot 2H_2O$	Merck	≥98%
Dimethylformamide (DMF)	C_3H_7NO	VWR HPLC Grade	99.99%
Toluene	C_7H_8	VWR HPLC Grade	99.99%
Trimethyl orthoformate	$C_4H_{10}O_3$	Acros Organics	98%

S2. Ligand Synthesis

Acetohydrazide and 2-methyl-1,3,4-oxadiazole were synthesized analogous to published procedures[1,2]. The ligands in their protonated state, 5-(3-methyl-4H-1,2,4-triazol-4-yl) isophthalic acid ($H_2{}^1L$ = H_2(Me-trz-ia)) and 3-(3-methyl-4H-1,2,4-triazol-4-yl) benzoic acid (H^2L = H(Me-trz-mba)), were prepared according to Lässig et al.[1]

S2.1 Synthesis of Acetohydrazide

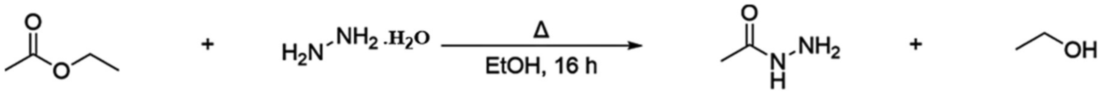

88.11 50.06 74.08 46.07

80 ml hydrazine monohydrate (1 eq, 82.0 g, 1.638 mol) together with 160 ml (1 eq, 144.3 g, 1.638 mol) of EtOAc was agitated in 300 ml of EtOH under reflux for 15 h. Under reduced pressure, the solvent and volatile components were evaporated, and the remaining mixture was then placed in the freezer to solidify. A colorless material was isolated by cold filtering and washing with EtOAc.

Yield: 58.67 g, 48 % of theory.

^{1}**H NMR (300 MHz, DMSO-d$_6$):** *δ*/**ppm** = 7.21 (s, 1H, N**H**); 3.93 (s, 2H, N**H**$_2$); 1.97 (s, 3H, C**H**$_3$)

S2.2 Synthesis of 2-Methyl-1,3,4-oxadiazole

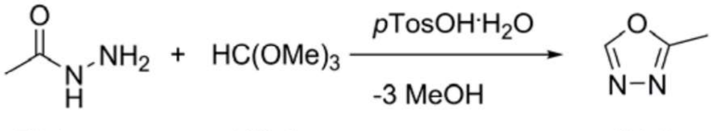

74,1 106,1 84,1

A mixture of 74.1 g (1.0 mol) acetohydrazide, 165 ml (160.1 g, 1.5 mol, 1.5 eq.) trimethyl orthoformate and 1.90 g (10.0 mmol, 0.01 eq.) p-toluenesulfonic acid monohydrate was heated to 80 °C then raised to 120°C respectively. The alcohol formed and the excess trialkyl orthoester were distilled off. The product was isolated as a colorless liquid by fractionated distillation at 10 mbar (75 °C).

Yield: 39.5 ml, 44.3 g, 62 % of theory.

^{1}**H NMR (300 MHz, DMSO-d$_6$):** *δ*/**ppm** = 8.26 (s, 1H, triazole-**H**); 2.32 (s, 3H, C**H**$_3$)

S2.3 Synthesis of 5-(3-Methyl-4H-1,2,4-triazole-4-yl) isophthalic acid ($H_2{}^1L$)

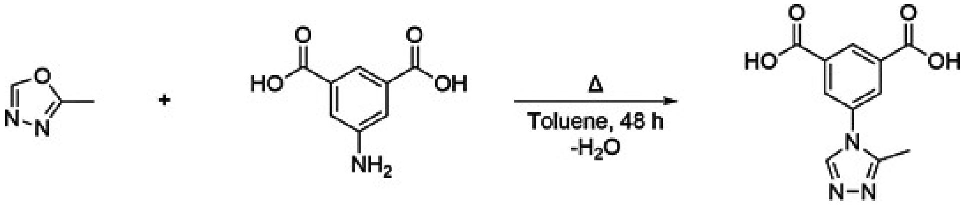

84.08 181.15 247.21

4.83 ml (5 eq, 5.42 g, 0.064 mol) of 2-methyl-1,3,4-oxadiazole and 2.35 g (1 eq, 0.013 mol) of 5-amino-1,3-isophthalic acid were mixed in 40 ml of toluene at 90 °C for 48 hours in N_2 atmosphere. The residual solid was stirred for 48 hours in DMF: EtOH (1:1 v/v) then filtered the product, or refluxed residual solid in the same solvent mixture for 30 min, then filtered while hot, resulting in a pure white product.

Yield: 1.52 g, 48 % of theory.

^{1}H NMR (300 MHz, DMSO-d$_6$): *δ*/**ppm** = 8.81 (s, 1H, triazole-**H**); 8.56 (t, 1H, isophthalate-**H**); 8.22 (d, 2H, ia-**H**); 2.37 (s, 3H, C**H**$_3$).

^{13}C NMR (100 MHz, DMSO-d$_6$): δ/ppm =165.9 (-C$_q$OOH); 150.3 (C$_q$-Me); 144 (triazole-CH); 134.9 (isophthalate -C$_q$-trz); 133.3 (C$_q$COOH); 130.1 (isophthalate -CH); 130 (isophthalate -CH); 10.6 (CH$_3$)

S2.4 Synthesis of 3-(3-Methyl-4*H*-1,2,4-triazole-4-yl) benzoic acid (H^2L)

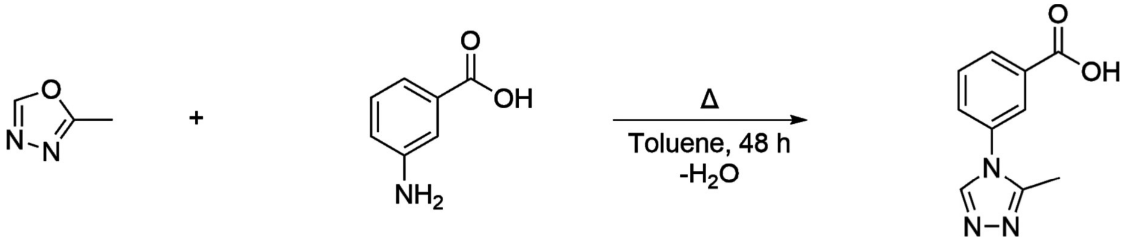

84.08 137.14 203.20

In inert atmosphere 2.75 g (1 eq, 0.020 mol) of 3-aminobenzoic acid and 7.50 ml (5 eq, 8.41 g, 0.100 mol) of 2-methyl-1,3,4-oxadiazole were stirred in 30 ml of toluene at 90 °C for 48 h. The crude product was filtered off, then refluxed with a mixture of water, ethanol and methanol (1:1:1; v/v/v) and filtered while hot. The product was dried on air to yield a white solid.

Yield: 2.317 g, 56 % of theory.

^{1}H NMR (300 MHz, DMSO-d$_6$): ***δ*/ppm** = 8.73 (s, 1H, triazole-**H**); 8.05 (dt, 1H, *m*-benzoate-**H**); 7.96 (t, 1H, *m*-benzoate -**H**); 7.78 (ddd, 1H, *m*-benzoate -**H**); 7.70 (t, 1H, *m*-benzoate -**H**); 2.33 (s, 3H, C**H**$_3$).

^{13}C NMR (100 MHz, DMSO-d$_6$): ***δ*/ppm** = 166.73 (-**C**$_q$OOH); 150.32 (triazole-**C**$_q$); 144.27 (trz-**C**H); 134.83 (*m*-benzoate-**C**$_q$N); 132.96 (*m*-benzoate -**C**$_q$COOH); 130.67 (*m*-benzoate -**C**H); 130.11 (*m*-benzoate -**C**H); 130.04 (*m*-benzoate -**C**H); 126.30 (*m*-benzoate -**C**H); 10.88 (-**C**H$_3$).

S3. Defect Engineered MOFs (DEMOF) Synthesis

The ReMOF $[Cu_2(^1L)_2]$ was synthesized from 0.1 mmol of linker ($H_2{}^1L$) and 0.1 mmol of copper chloride dihydrate. The DEMOFs $[Cu_2(^1L_{(1-x)}{}^2L_x)_2]$ were synthesized by replacing some (x) of $H_2{}^1L$ by H^2L keeping the molar ratio of Cu:L at 1:1.

Solvothermal synthesis: A stainless steel autoclave with Teflon inset (PARR) was loaded with $H_2{}^1L$, H^2L, copper chloride dihydrate and 10 ml solvent (DMF:EtOH), sealed and the reaction mixture was heated within 1 h up to 120 °C. The temperature was kept on a constant level for 5 h, then the autoclave was cooled slowly to room temperature during a period of 60 h**.**

Table S1: Molar ratios for the synthesis of the ReMOF $[Cu_2(^1L)_2]$ and DEMOFs $[Cu_2(^1L_{(1-x)}{}^2L_x)_2]$

Name of sample	Molar Ratio ($H_2{}^1L$: H^2L)	$H_2{}^1L$	H^2L	$CuCl_2 \cdot 2H_2O$	Yield
ReMOF	100:0	24.7 mg	-	17.1 mg	5.2 mg
DEMOF_2.9%	92:8	22.73 mg	1.62 mg	17.1 mg	4.25 mg
DEMOF_7.0%	80:20	19.76 mg	4.06 mg	17.1 mg	4.75 mg

Colour change in DEMOFs with increasing content of $^2L^-$

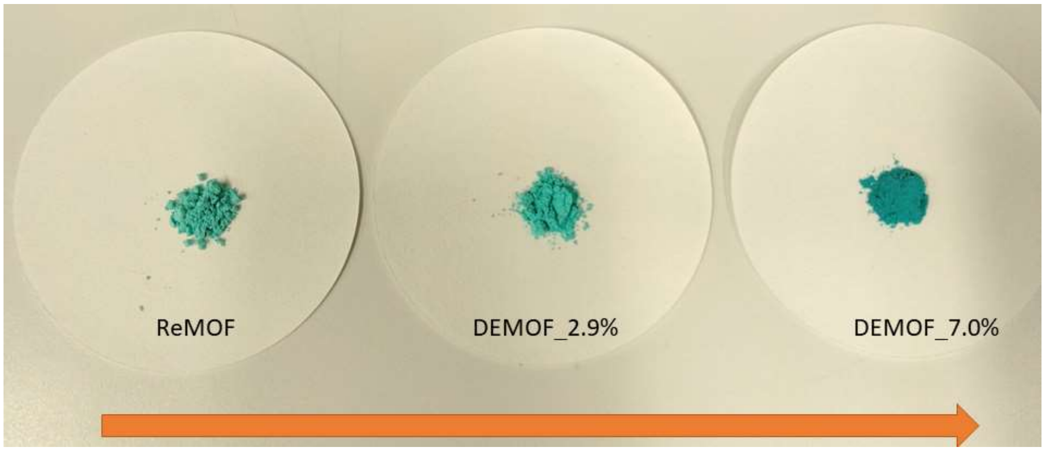

Figure S1. Colour variation with increasing percentage of $^2L^-$ in the reaction mixture. Left to right: ReMOF (green), DEMOFs with x=2.90% and x=7.07% (bluish-green).

The naming convention used in this manuscript describes DEMOF samples based on the molar ratio of linkers they contain within their framework, such as DEMOF_2.9% and DEMOF_7.0% (see **Sections S8** and **S9** for determination of the percentages of incorporated linkers). In order to have defined conditions, the MOF samples were solvent exchanged with MeOH and stored in glass vials under MeOH. For measurements, the solvent was removed and the sample was activated in vacuum.

S4. Single crystal structure of ReMOF [$Cu_2(^1L)_2$]

The results of the single crystal X-ray diffraction analysis were reported by Kobalz et al.[2]

Formula: $C_{22}H_{14}N_6O_8Cu_2$, Molecular Mass: 617.47 g mol^{-1}

Space group $P2_1/c$ (No. 14),

Unit cell parameters at 180 K: a = 10.859(1), b = 12.786(2), c = 14.026(2) Å, β = 110.508(8)°, V = 1823.9(4) Å^3, Z = 2.

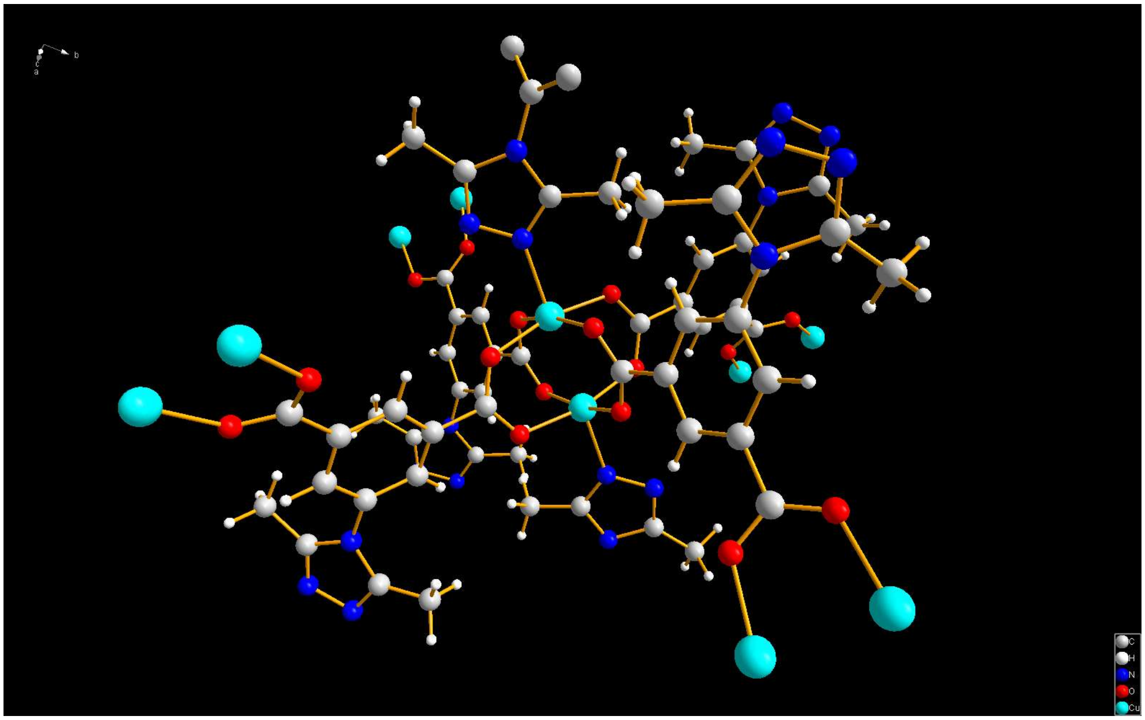

Figure S2. Fragment of the crystal structure of ReMOF (as synthesized) showing the coordination of the ligand $^1L^{2-}$ and the paddle wheel unit as a six-fold connecting node.

S5. Powder X-ray Diffraction

S5.1 Powder X-ray diffraction (PXRD) of ReMOF and DEMOFs

PXRD patterns were obtained using a STOE STADI-P diffractometer with Cu-$K_{\alpha1}$ radiation (λ = 1.54060 Å). The samples for these measurements were prepared in glass capillaries (Hilgenberg, outer diameters 0.3, 0.5 or 0.7 mm) and measured at r.t.

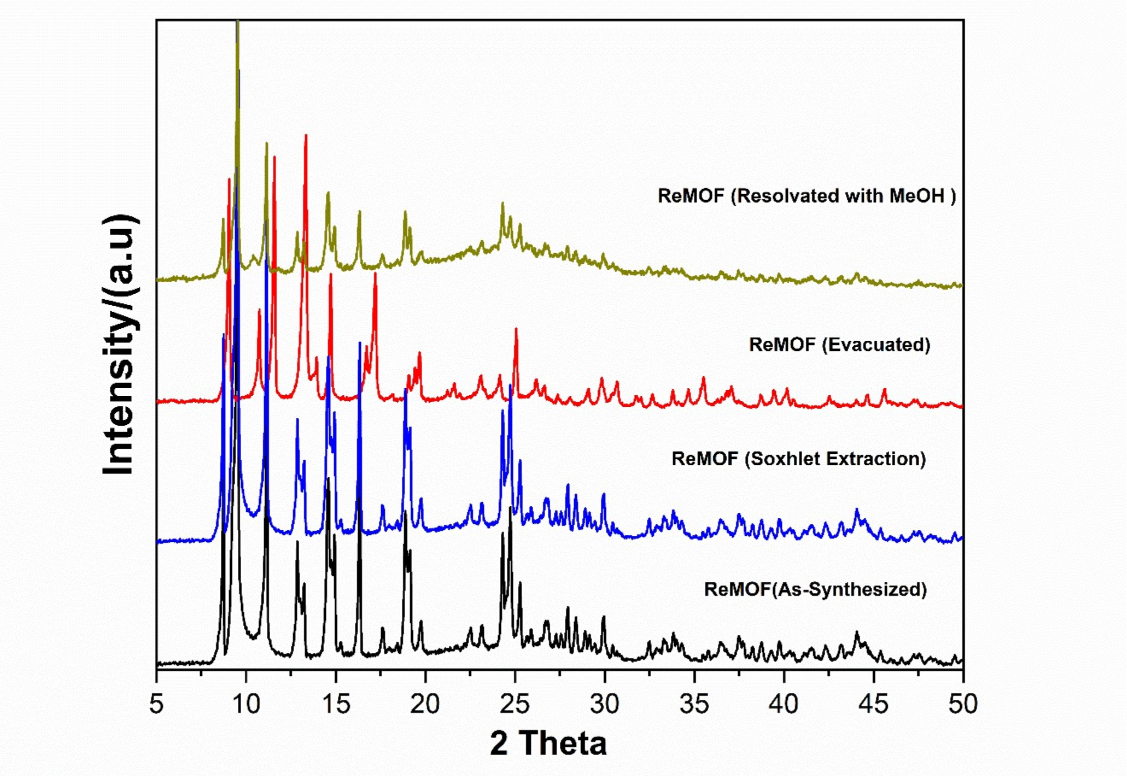

Figure S3. PXRD patterns of the ReMOF [$Cu_2(^1L)_2$] after various treatments.

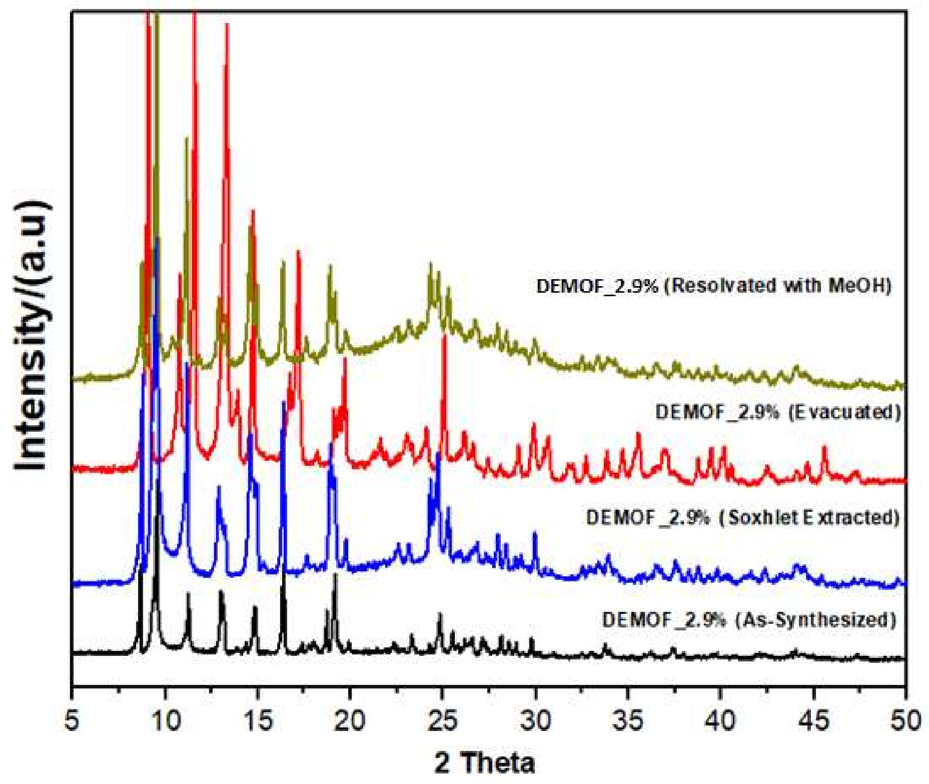

Figure S4. PXRD patterns of DEMOF_2.9% after various treatments.

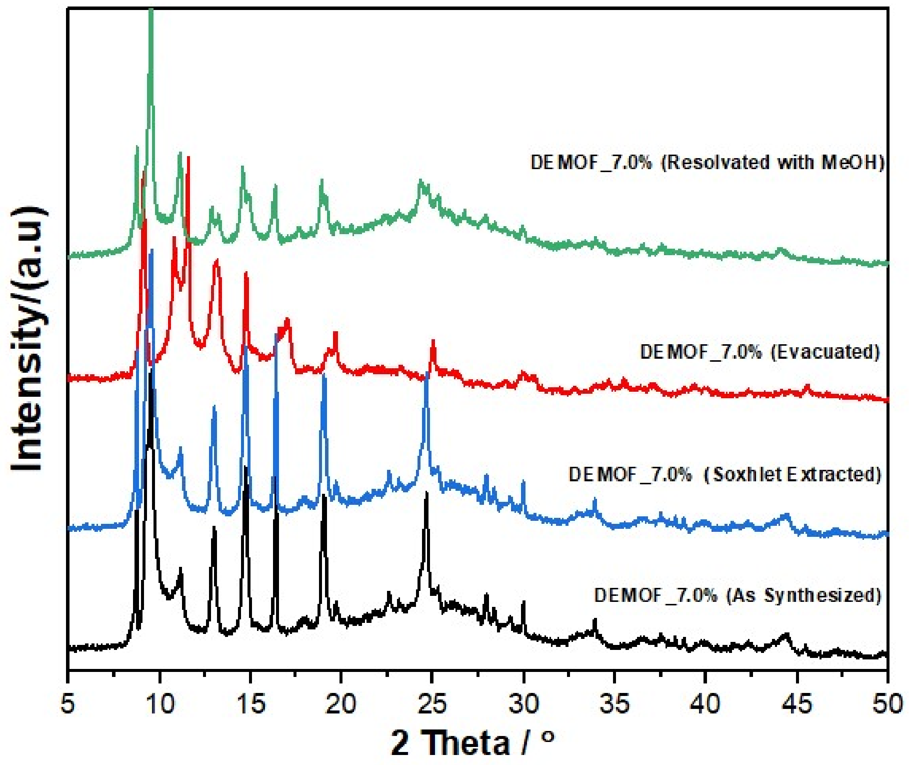

Figure S5. PXRD patterns of DEMOF_7.0% after various treatments.

S5.2 Strain effect

Micro-strain broadening[3] effects in the PXRD patterns of the synthesized MOFs were considered by extracting the ε_0 and β(FWHM(Strain)) values using TOPAS (Bruker AXS) software[4] from the broadest peak identified in PXRD.

PXRD data are influenced by **defects** such as displacements, vacancies, interstitials, substitutions, and related effects. Microstrain can be conceived by considering two extreme values of the lattice spacing d, namely d + Δd and d – Δd, where $\varepsilon_0 = \Delta d/d$ represents the relative "mean" deviation (more precise: 50% probability of the undistorted state). We considered the most intense and highly broadened peak around 2Θ = 9.4° for the microstrain determination.

$$\boldsymbol{\varepsilon_0 = \frac{\beta_{FWHM(Strain)}}{4tan\theta}}$$

Here, $\beta_{FWHM(Strain)}$ represents the full width at half maximum of the line profile component related to the microstrain broadening.

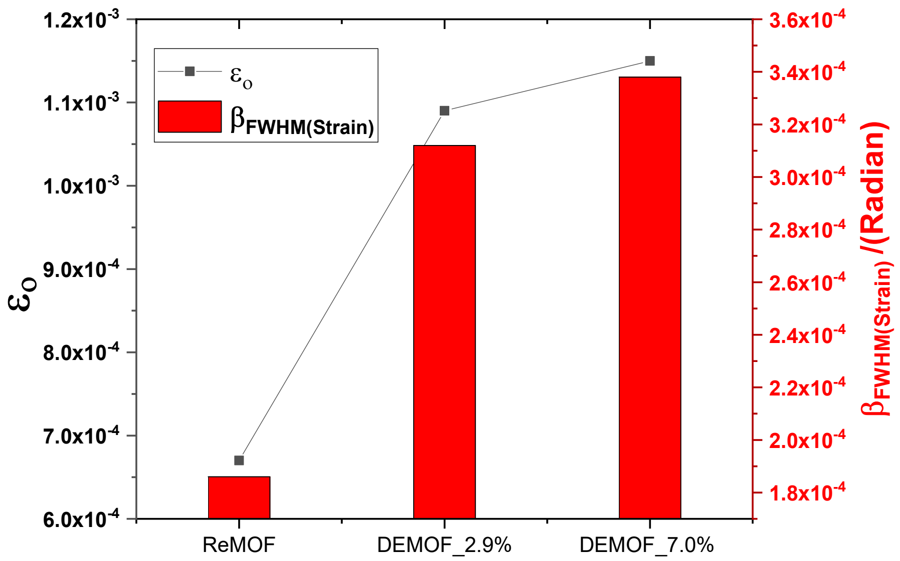

Figure S6. Variation of ε^0 and $\beta_{FWHM(Strain)}$ with increasing $^2L^-$ percentage in the DEMOF samples.

S5.3 Pawley Refinement

For the Pawley refinement of ReMOF, DEMOF_2.9% and DEMOF_7.0%, the crystalline structure of the ReMOF[2] was used as a starting model. The Pawley refinement was carried out using TOPAS[4] (Bruker AXS). The experimental data and crystal data for ReMOF and DEMOF samples are listed in the figures below. All the peaks are matching with the simulated ones which indicates the ReMOF and DEMOF samples are phase pure. With increasing content of $^{2}L^{-}$, an increase in line broadening is observed.

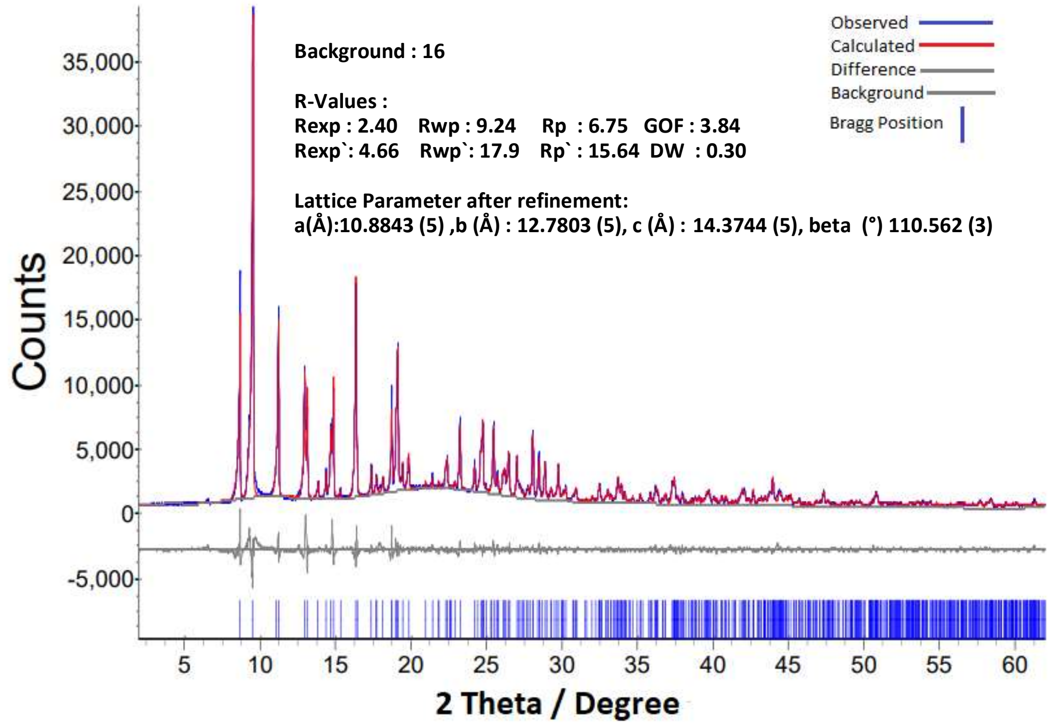

Figure S7. Pawley refinement of ReMOF

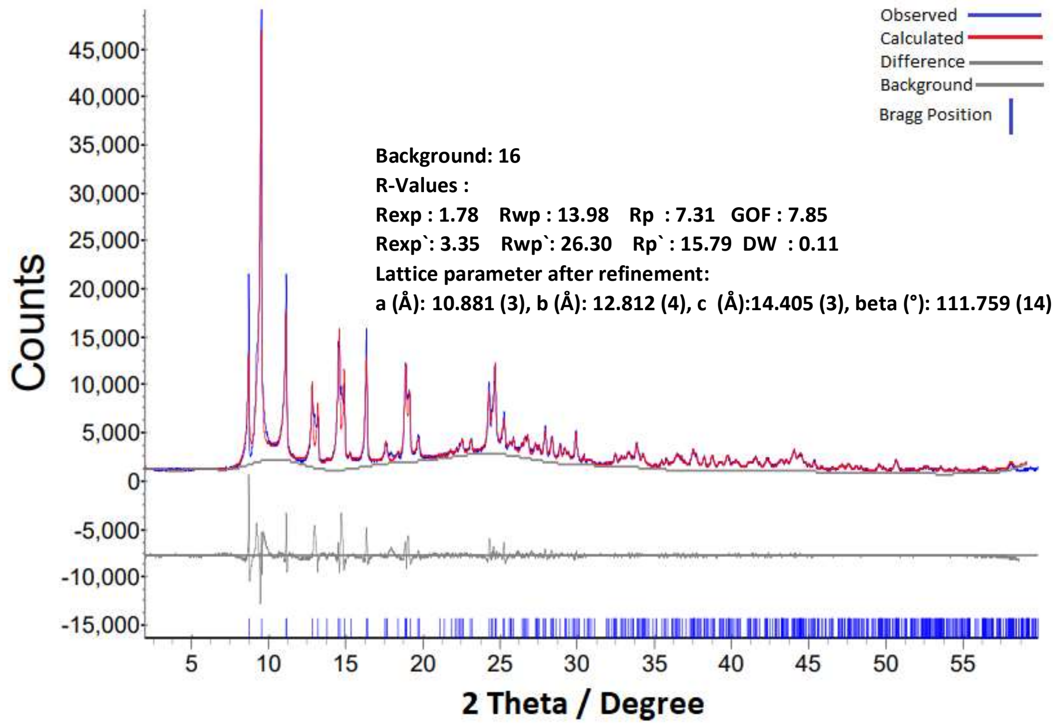

Figure S8. Pawley refinement of DEMOF_2.9%

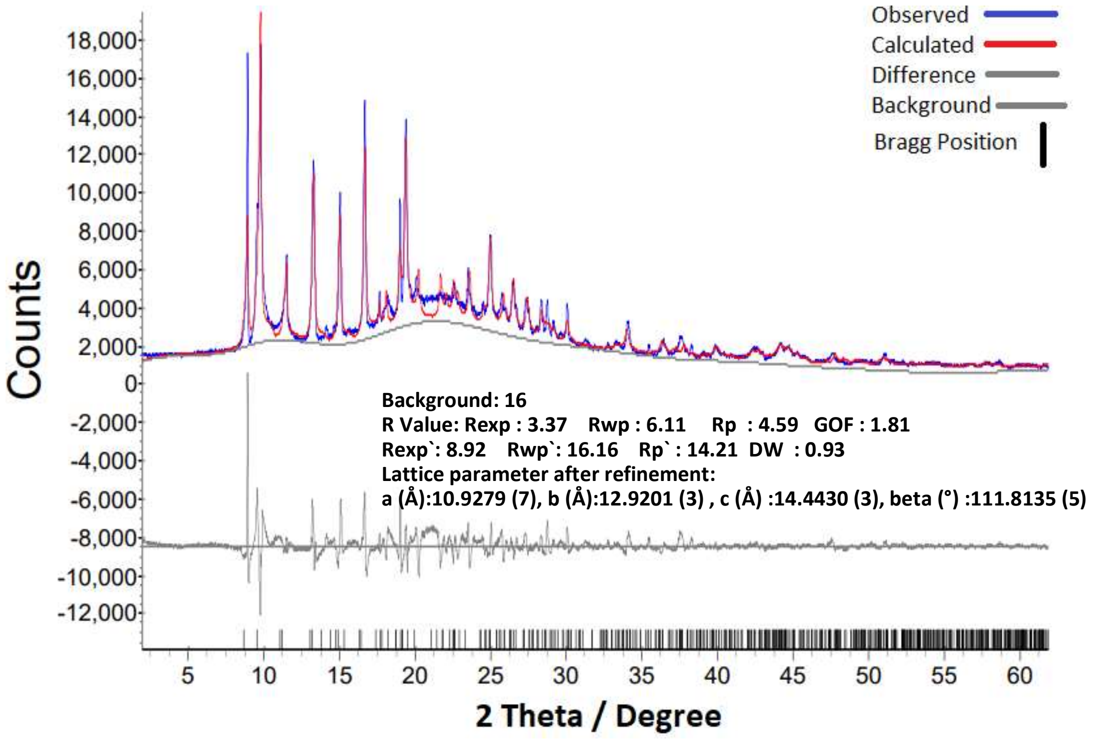

Figure S9. Pawley refinement of DEMOF_7.0%

S6. FTIR Spectroscopy

The infrared spectra were recorded with a Bruker Vertex 80v FTIR spectrometer utilizing OPUS 6.0 (Bruker) for analysis. All samples were prepared as KBr pellets from 2 mg of MOF sample and 200 mg of KBr. For further analysis, the samples were measured before and after treatment at 100 °C for removal of water molecules. Normalization and baseline correction were done after spectra acquisition.

The bands at 473 and 726 cm^{-1} are assigned to Cu-O bending and stretching vibrations, respectively. This agrees well with references,[5,6] where those vibrations are assigned to the Cu–O group. The band around 1450 cm^{-1} belongs to combination of benzene ring stretching and deformation modes. Bands between 1300-1500 cm^{-1} are attributed to symmetric stretching vibrations and 1500-1700 cm^{-1} to antisymmetric stretching vibrations of the carboxylate groups. The band at 675-814 cm^{-1} is probably the aromatic out of plane (oop) bending vibration for aromatic C-H. Two emerging peaks at 560 cm^{-1} and 1000 cm^{-1}, attributed to (CCC) in-plane bending and twisting ring modes[78], are not significant in ReMOF but become well distinct after $^{2}L^{-}$ incorporation.

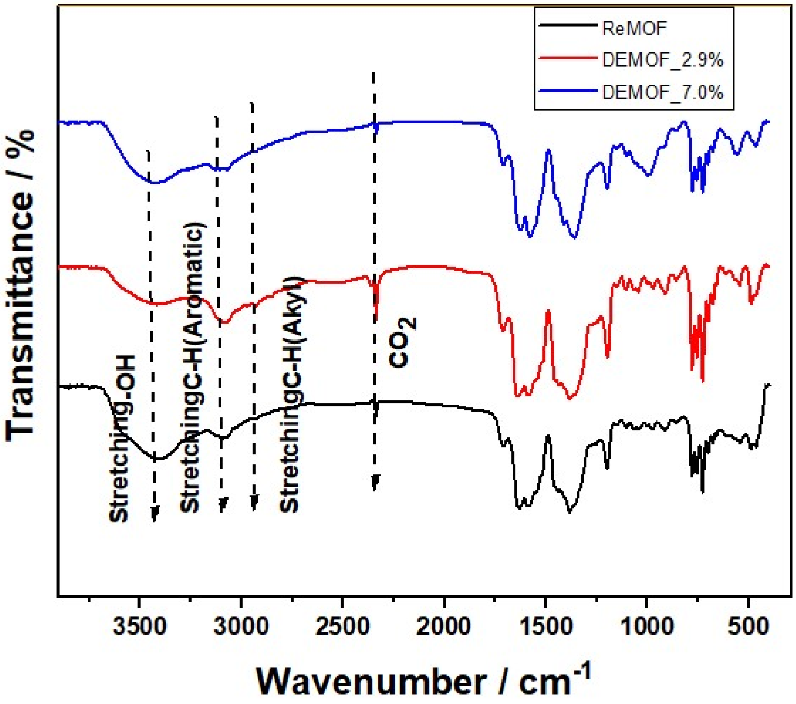

Figure S10. Stacked FTIR spectra of ReMOF, DEMOF_2.9% and DEMOF_7.0%.

S7. Raman Spectroscopy

The Raman spectra (**Figure 2(b)**, main manuscript) were acquired on a confocal modular Raman measurement setup (S&I Spectroscopy & Imaging GmbH, Warstein, Germany) equipped with a 532 nm excitation laser (Cobolt AB, Solna, Sweden) with a power of 100 mW. For Raman measurements, the sample was positioned on an IX71 epifluorescence upright microscope (Olympus Corporation, Tokyo Japan), which was equipped with a LUCPlanFI 40-fold objective (NA 0.6, Olympus Corporation, Tokyo Japan) for focusing the laser beam onto the sample. The incident (excitation) laser power on the sample was approx 5-6 mW, controlled using a ND (Neutral-density) filter wheel (Thorlabs INC, NJ, USA). The scattered light passed an Andor Kymera 193i Spectrograph (Oxford Instruments, Abingdon, United Kingdom) with an entrance slit of 100 µm and a grating of 1800 lines/mm and was detected via an Andor Newton 1024 x 255 CCD camera (Oxford Instruments, Abingdon, United Kingdom). An acquisition time of 1 s was used for all measurements. The spectra were collected in the range of 190 to 1900 cm^{-1} with a spectral resolution of 3 cm^{-1}. VistaControl V4.2 Build 12596 (S&I Spectroscopy & Imaging GmbH, Warstein, Germany) was used as interface and recording software[9].

According to the spectrum, Cu^{2+} ion-related vibrational modes cause peaks up to 600 cm^{-1}. Cu-Cu dimer moieties are responsible for the stretching mode that causes the peaks from 190 to 270 cm^{-1}. An insignificant peak at 400-490 cm^{-1} is attributed to the Cu-O stretching mode of the coordinated carboxylate bridges. The C-H bending vibration modes are seen at 756 cm^{-1} and 866 cm^{-1}, respectively. The symmetric stretching mode of C=C moieties can be seen at about 1012 cm^{-1}. The vibrational mode of carboxylate groups is responsible for the region of 1400 to 1600 cm^{-1}. The peaks around 1389 cm^{-1} belong to C-N moieties[10] of the triazole group. All the Raman spectra in the current study very well match with those of HKUST-1 type MOFs.[7]

S8. NMR Spectroscopy

An Avance DPX-300 NMR spectrometer (Bruker) was used to record the solution phase ^{1}H NMR spectra (300 MHz). For determining the ratio $^1L^{2-}$: $^1L^-$ in the DEMOFs $[Cu_2(^1L_{(1-x)}{}^2L_x)_2]$, the MOFs are digested using deuterated digestion media (1M NaOH in D_2O) prior to ^{1}H NMR analysis. The solvent D_2O was used as internal lock, the spectra were referenced to D_2O 4.79 ppm (reference peak is omitted for the better visualization of spectra). Data acquisition was performed with a delay time of 2 to 2.5 s. Liquid solution ^{1}H NMR spectroscopy allows for the quantitative analysis and elucidation of the organic components such as the linker, modulator, and pore filling fluids (as a molar ratio with other organic components).

Before digestion, all samples were thoroughly washed with DMF: EtOH and subsequently went through solvent exchange and evacuation up to 7 days, i.e., the samples are fully activated without any solvent inside the pore. For determination of the ratio $^1L^{2-}$: $^2L^-$ in the MOFs, 20 mg of the mixed linker MOFs was dissolved in 0.7 ml of a digestive medium containing NaOH (1M) in D_2O in a test-tube. The test-tube was sealed, kept for sonication for 2 h and then left to digest for 24 hours. The inorganic component of the ReMOF and DEMOFs precipitates as CuO, which was removed by filtration using a Whatman filter paper (glass microfiber). The organic portion of the MOF (linkers and solvent if still left after activation) was transferred to an NMR tube. The molar ratios between the protons can be determined by integration of the corresponding NMR signals. Specific ^{1}H NMR peaks of $^2L^-$ after digestion are located at δ/ppm = 8.480 (s, 1H, triazole-H); 7.92 (dt, 1H, m-benzoate-H); 7.76 (t, 1H, m-benzoate -H); 7.52-7.57 (ddd, 1H, m-benzoate -H); 7.43-7.45 (dt, 1H, m-benzoate -H); 2.30 (s, 3H, CH_3-triazole). We determined the molar fraction of $^2L^-$ within the dissolved linker mixture of $^1L^{2-}$ and $^2L^-$ by following the procedure described in a previous paper[11]: I_{1_L} and I_{2_L} are the integrated ^{1}H NMR intensities of specific protons of $^1L^{2-}$ and $^2L^-$ in the ^{1}H NMR spectra, N_{1_L} and N_{2_L} are the corresponding numbers of equivalent hydrogen atoms. Peaks around 2.30 (s, 3H, C**H**$_3$-triazole) from $^2L^-$ and 2.35 (s, 3H, C**H**$_3$-triazole) from $^1L^-$ have been utilized for this integration.

$$\textbf{Doping ratio} = ((I_{2_L}/N^2{}_L) / (I_{2_L}/N^2{}_L + I_{1_L}/N^1{}_L)) \times 100\%$$

Table S2: Peak integration and ratios of the amount of $^2L^-$ to that of total linkers in the obtained DEMOFs.

**Sample**	I_{2_L}	I_{1_L}	**Molar Ratio of $^2L^-$ / $^1L^{2-}$ + $^2L^-$**	
			Percentage of H^2L in synthesis	Percentage of $^2L^-$ in DEMOF
ReMOF	0	100	0	0
DEMOF_2.9%	3	84.20	8%	3.44%
DEMOF_7.0%	3	35.29	20%	7.38%

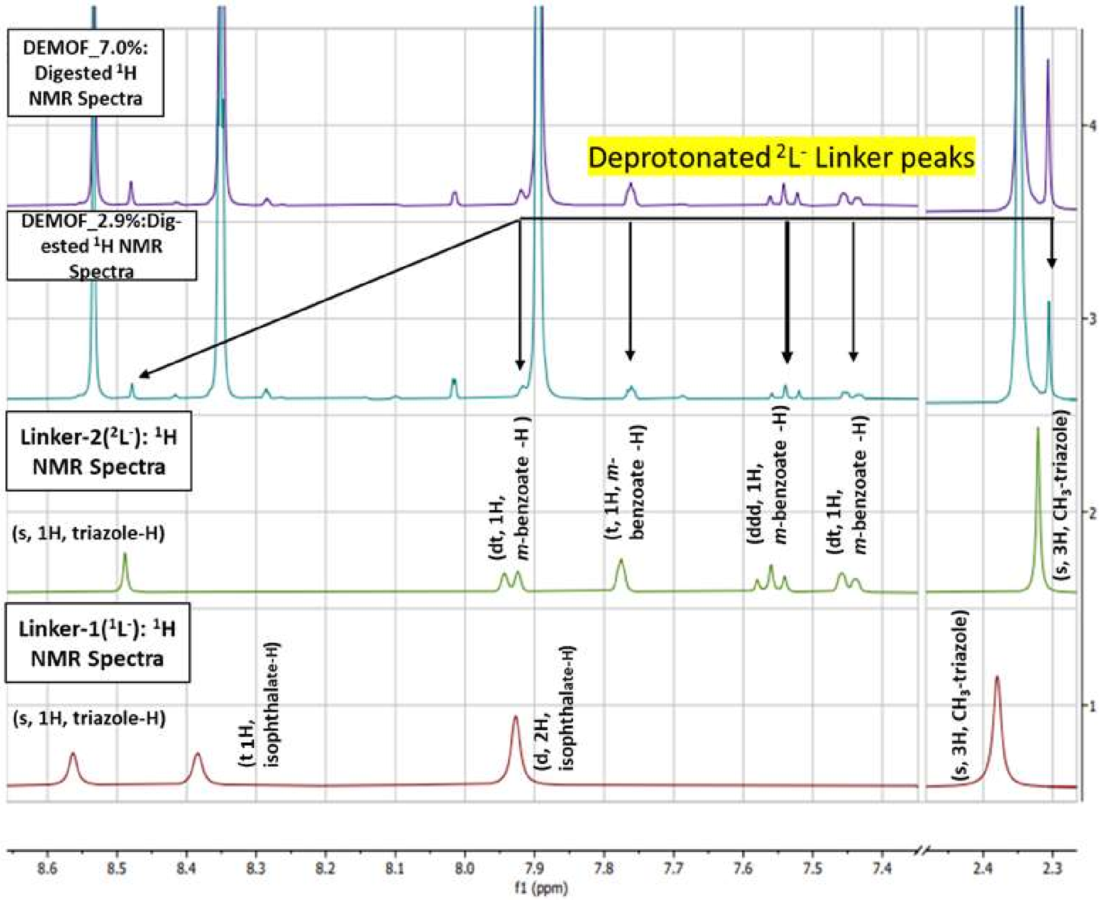

Figure S11. ^{1}H NMR spectra of digested DEMOFs: corresponding peaks of $^{1}L^{2-}$, $^{2}L^{-}$, DEMOF_2.9% and DEMOF_7.0%.

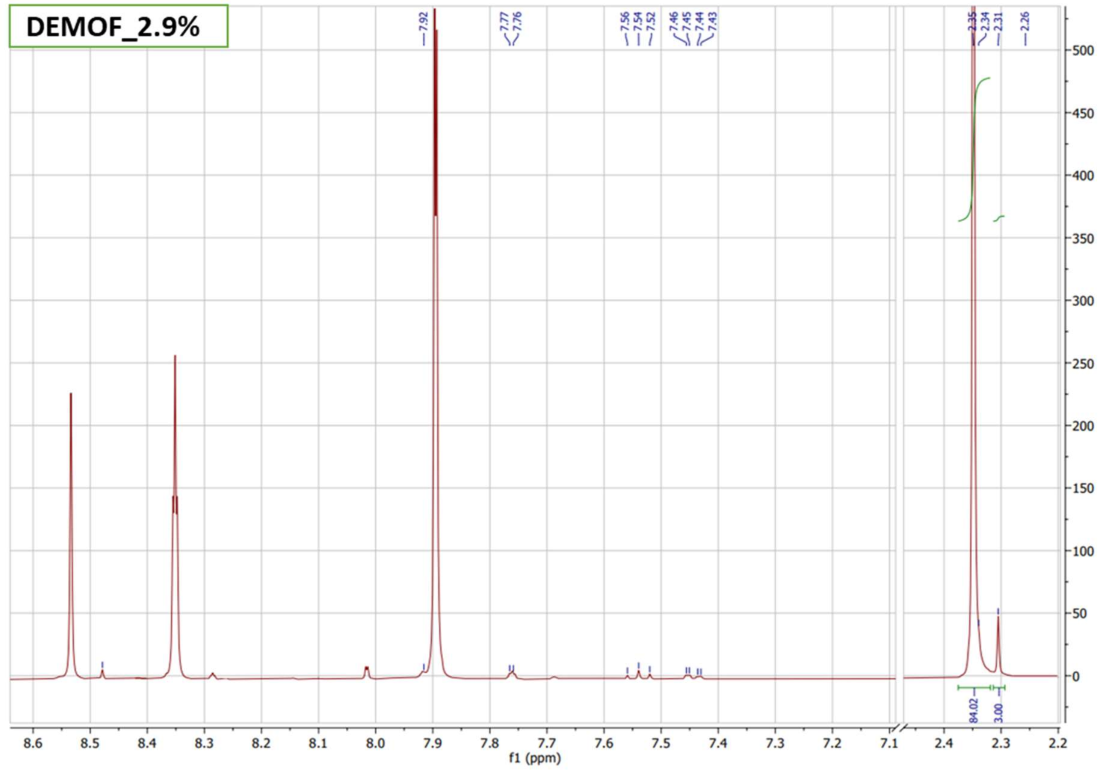

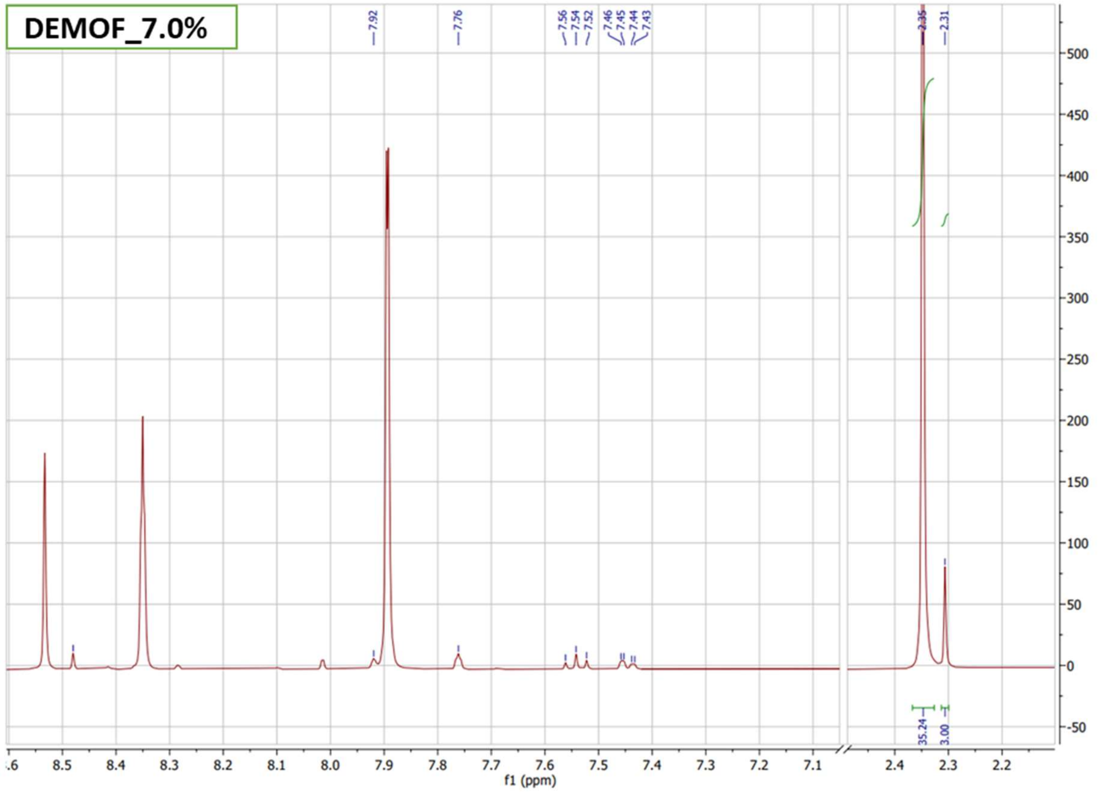

Figure S12. ^{1}H NMR spectra and peak intensities of digested DEMOF_2.9% (top) and DEMOF_7.0% (bottom).

S9. HPLC Analysis

Experimental setup:

HPLC System Agilent 1290 Infinity 2, Detector: DEBAV3450

Buffer: 15mM $NH_4^+HCOO^-$, pH 7.9

Detection: Peak area measured at 254 nm.

Column: ET 250/8/4 NUCLEOSIL, 5 NH_2, 250 mm, 5 µm particles

Calibration curve: To get the appropriate calibration curves and the defined amounts of $^2L^-$ in a particular DEMOFs sample, the absorption of organic linkers associated with 6 different concentrations was measured and calibrated by determining the respective peak area.

The DEMOFs were synthesized with 8%, 12%,16%, 20% and 24% of defective linker (H^2L) in the reaction mixture. For sample preparation, 5 mg of the DEMOF were dissolved in 3 ml 0.5M NaOH (aq) under sonication, the solution was filtered, diluted, and samples of the stock solution (in ACN: H_2O, 50:50) were analysed by injecting 5 µL of the respective solution into an NH_2 based column. Separation of $^1L^{2-}$ and $^2L^-$ is done by hydrophilic interaction chromatography (HILIC) under isocratic conditions.[19]

Table S3: HPLC analysis of digested MOFs.

**Samples**	**$^1L^{2-}$ (Regular Linker)**	**$^2L^-$ (Def. Linker)**	**Mass % $^2L^-$ in total linker ($^1L^{2-}$+$^2L^-$)**	**Mole % $^2L^-$ in total linker ($^1L^{2-}$+$^2L^-$)**
**ReMOF**	0.647 mg/mL	-	-	-
**DEMOF_2.9%**	0.557 mg/mL	0.014 mg/mL	2.4 %	2.9%
**DEMOF_4.5%**	0.668 mg/mL	0.026 mg/mL	3.7 %	4.5%
**DEMOF_6.5%**	0.726 mg/mL	0.042 mg/mL	5.4%	6.5%
**DEMOF_7.0%**	0.677 mg/mL	0.042 mg/mL	5.8 %	7.0%
**DEMOF_8.6%**	0.694 mg/mL	0.054 mg/mL	7.2%	8.6%

S10. Simultaneous Thermal Analysis (TG-MS)

The TG-MS analyses were carried out using corundum crucibles on a thermobalance STA 449 F1 Jupiter (Netzsch)[12] coupled to an Aeolos QMS 403C mass spectrometer. The sample was heated at a rate of 10 K min^{-1} up to 900 °C under constant flow of Argon (99.999 %). All samples were Solvent exchange with methanol to achieve complete solvent exchange. Prior to each measurement the sample was evacuated at room temperature in the instrument, however, the crucibles had contact to air when transferred to the sample holder in the instrument. This might lead to adsorption of minor amounts of water, resulting in an initial weight loss.

Initial Mass Loss: Before the decomposition phase, there is a minor mass loss which might correspond to the desorption of weakly bound molecules such as water or residual solvents. The ReMOF sample begins to decompose at 289.7 °C (onset temperature) and this decomposition process concludes at 313.9 °C. The total mass change during this phase suggests decomposition of the organic components. DEMOF_2.9% shows a similar pattern, with decomposition starting at a slightly lower temperature of 284.6 °C and ending at 312.7 °C. DEMOF_7.0% starts decomposing at an even lower temperature of 279.8 °C and ends at 293.1 °C, indicating that $^{2}L^{-}$ incorporation affects its thermal stability.

The decomposing temperature range in each sample is critical as it provides insight into the thermal stability. The lower onset temperatures in the DEMOFs sample compared to the parent ReMOF could be due to a higher defect concentration, which leads to lower thermal stability. Similar results were also noticed by Fang et al.[11] in defect engineered HKUST-1 MOFs with varied linker incorporation. Increasing the defective linker concentration leads to decreasing onset temperatures compared to parent HKUST-1 MOF.

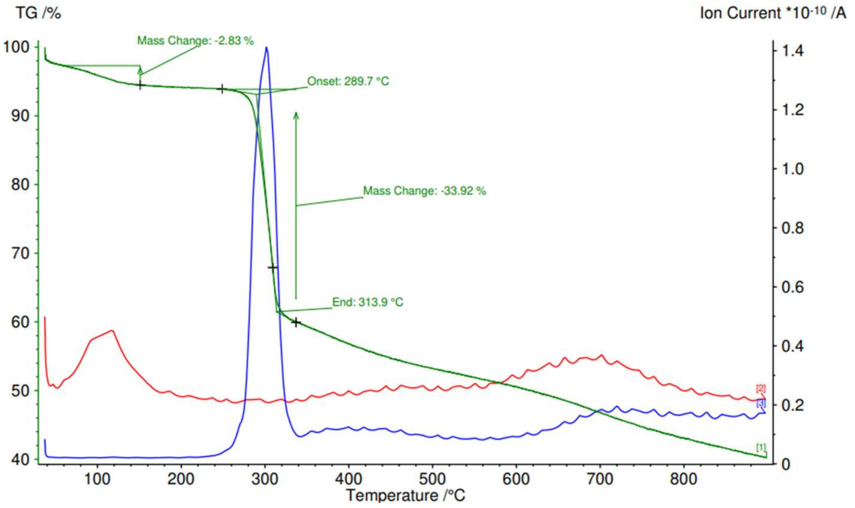

Figure S13. TG-MS of ReMOF [$Cu_2(^{1}L)_2$]. MS-Signals of $(H_2O)^+$ (m/z=18, red) and $(CO_2)^+$ (m/z=44, blue) illustrate the evaporation of guest molecules and decomposition of the framework, respectively.

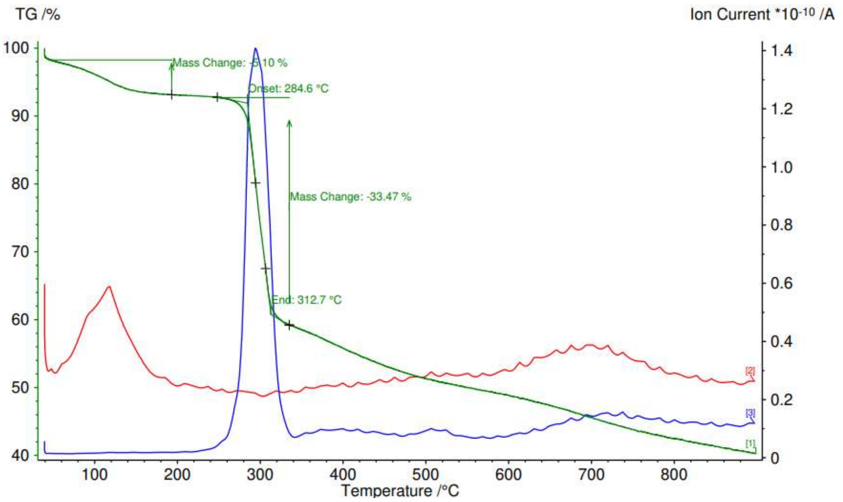

Figure S14. TG-MS of DEMOF_2.9%. MS-Signals of $(H_2O)^+$ (m/z=18, red) and $(CO_2)^+$ (m/z=44, blue) illustrate the evaporation of guest molecules and decomposition of the framework, respectively.

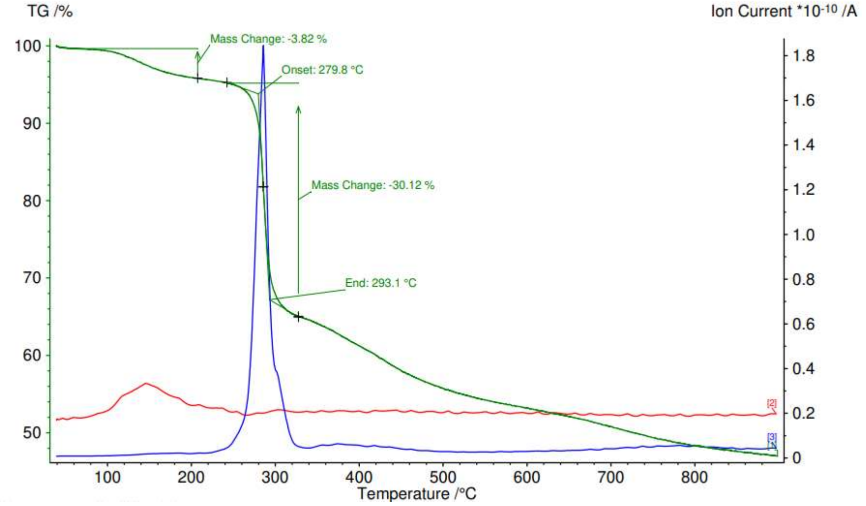

Figure S15. TG-MS of DEMOF_7.0%. MS-Signals of $(H_2O)^+$ (m/z=18, red) and $(CO_2)^+$ (m/z=44, blue) illustrate the evaporation of guest molecules and decomposition of the framework, respectively.

S11. X-ray Photoelectron Spectroscopy (XPS) X-ray Photoelectron Spectroscopy**:** In order to distinguish between different oxidation states. Cu^{I} and Cu^{II}, in the ReMOF and DEMOF samples,

Instrumentation details: X-ray photoelectron spectroscopy (XPS) analysis was conducted using a Thermo Fisher Scientific K Alpha+ XPS system (Thermo Fisher Scientific Instruments, UK). For the measurements, monochromatic Al-K_{α} radiation was utilized, generated in a sealed X-ray tube with a beam current of 6 mA and an acceleration voltage of 12 kV. Instrument calibration was verified using the Ag 3d peak at 352 eV. The spot size for analysis on the sample was 400 micrometers. Binding energies were referenced to the C1s peak at 284.8 eV.

All spectra were calibrated with respect to adventitious carbon observed at 285 eV. A survey scan was performed on each sample before the elemental scans in order to identify every element that was present. Using the CASA XPS tool[13], the XPS spectra were deconvoluted using a Gaussian Lorentzian mix function with smooth linear background subtraction.

In all the XPS survey scans for ReMOF and DEMOF samples, the spectra display the distinctive peaks from copper, Cu LMM Auger, oxygen, nitrogen, and carbon for each sample. The accompanying Cu LMM Auger peaks at 916.5 eV, typical for Cu^{I}-Cu^{II} pairs and at 917.2 eV[18], typical for Cu^{II}-Cu^{II} species, provide additional evidence that two different types of dinuclear units are present in Cu LMM Auger spectra.

XPS survey spectra of all ReMOF and DEMOF samples show C-1s, O-1s, N-1s contributions as expected from the 1,2,4-triazolyl isophthalate and benzoate linkers.

Table S4: XPS binding energy for different elements as reference from literature[11, 14–16, 20].

XPS spectrum	Component	Binding energy [eV]
Cu $2p_{3/2}$	Cu^{I}	933.2 ± 0.5
	Cu^{II}	935.1 ± 0.5
	Satellite 1	940.3 ± 0.5
	Satellite 2	944.5 ± 0.3
Cu LMM Auger	Cu^{I}	916.2 ± 0.5
	Cu^{II}	917.7 ± 0.5

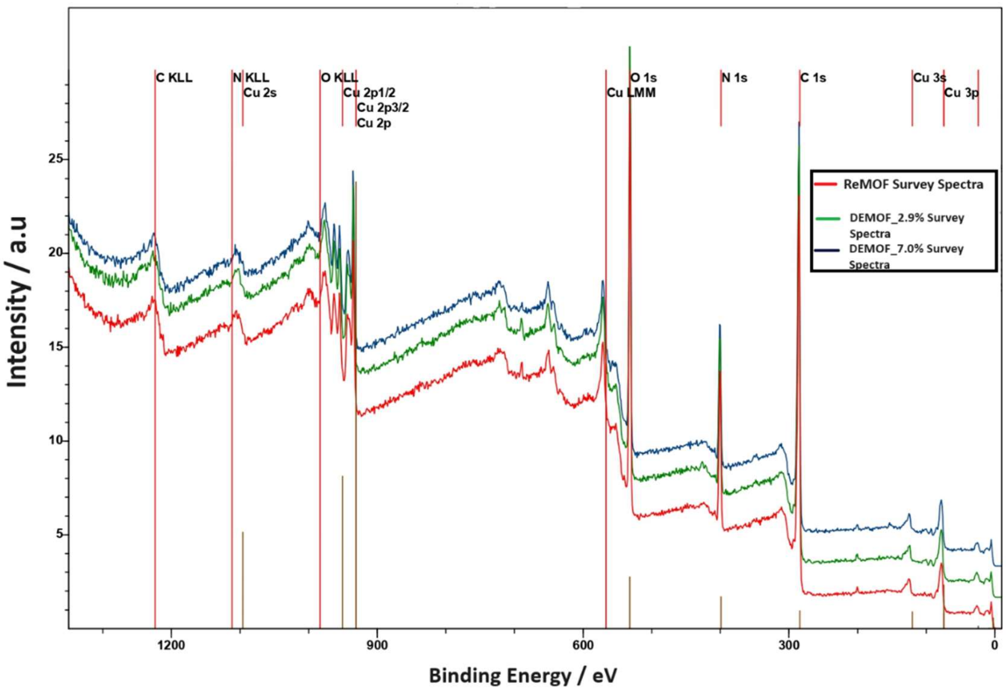

Figure S16. XPS survey spectra of ReMOF, DEMOF_2.9% and DEMOF_7.0%

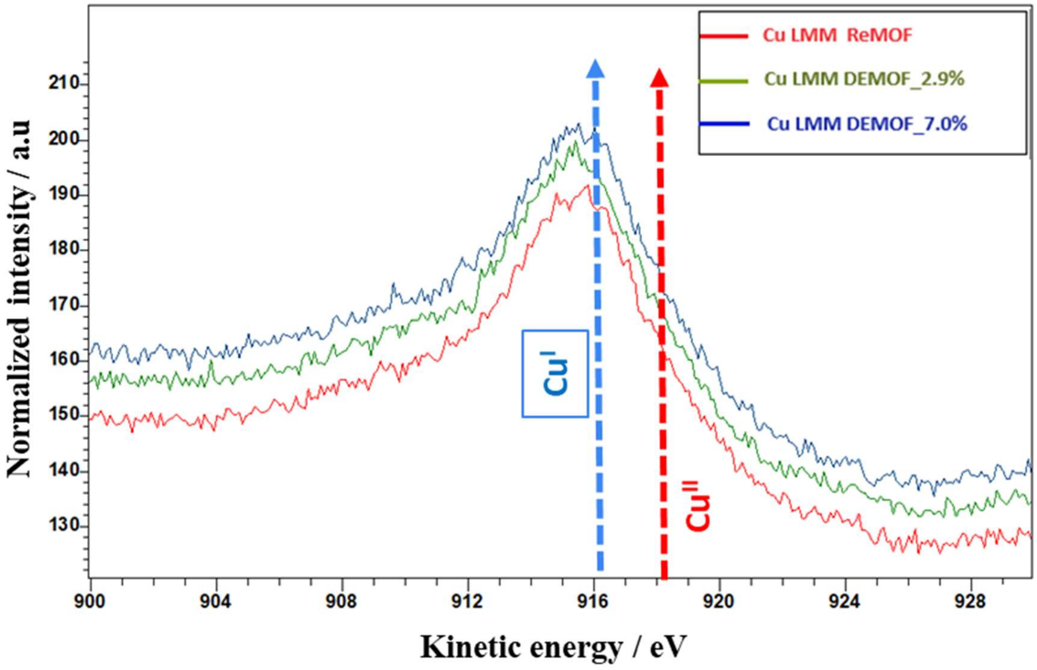

Figure S17. XPS Cu LMM spectra of ReMOF, DEMOF_2.9% and DEMOF_7.0%.

S12. Electron Paramagnetic Resonance (EPR)

Sample Preparation: Approximately 25 mg of as-synthesized ReMOF and DEMOF samples in methanol were placed inside the quartz tube (inner diameter 3.8 mm). EPR studies for all ReMOF and DEMOFs were carried out at solvated state, i.e., in contact to methanol.

cw EPR Measurements: A series of X-band and Q-band *cw* EPR measurements were conducted using the commercial spectrometers EMXmicro and EMX 10-40 (Bruker), respectively. The X-band *cw* EPR spectra were measured at temperatures ranging from T = 10 K to T = 160 K by means of a Bruker EMXmicro spectrometer fitted with a Bruker ER4119HS cylindrical cavity and the He cryostat ESR900 (Oxford instruments) while in case of Q band measurements, the spectrometer was fitted with a cylindrical cavity and an CF935 cryostat (Oxford Instruments) with a working temperature range between T = 20 K and 295 K. The microwave power and modulation amplitude were adjusted such that minimum signal distortion can be obtained. EPR spectral analysis was performed the Easyspin numerical simulation package for MATLAB[17] .

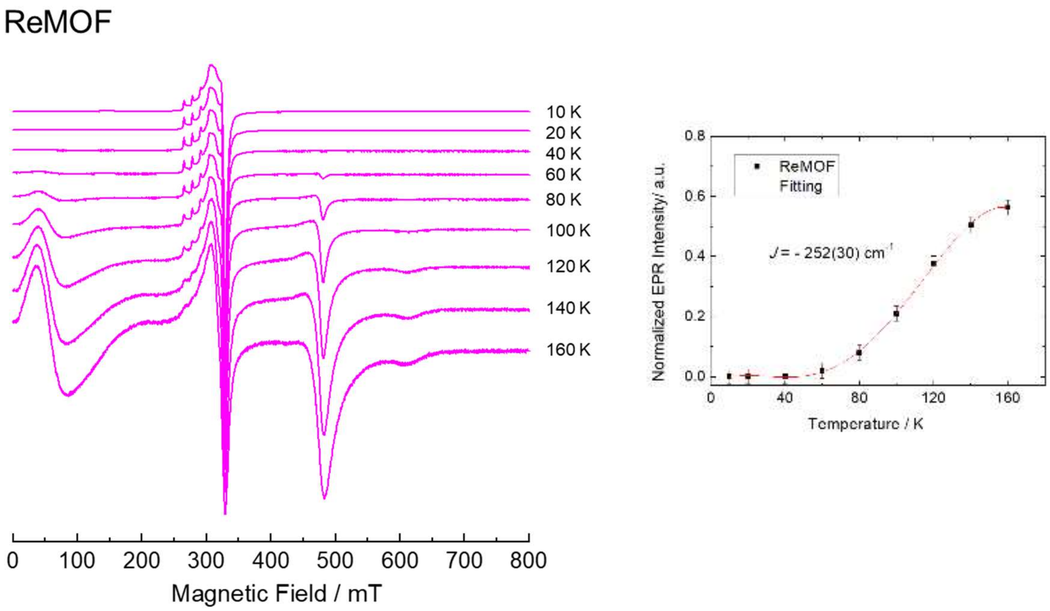

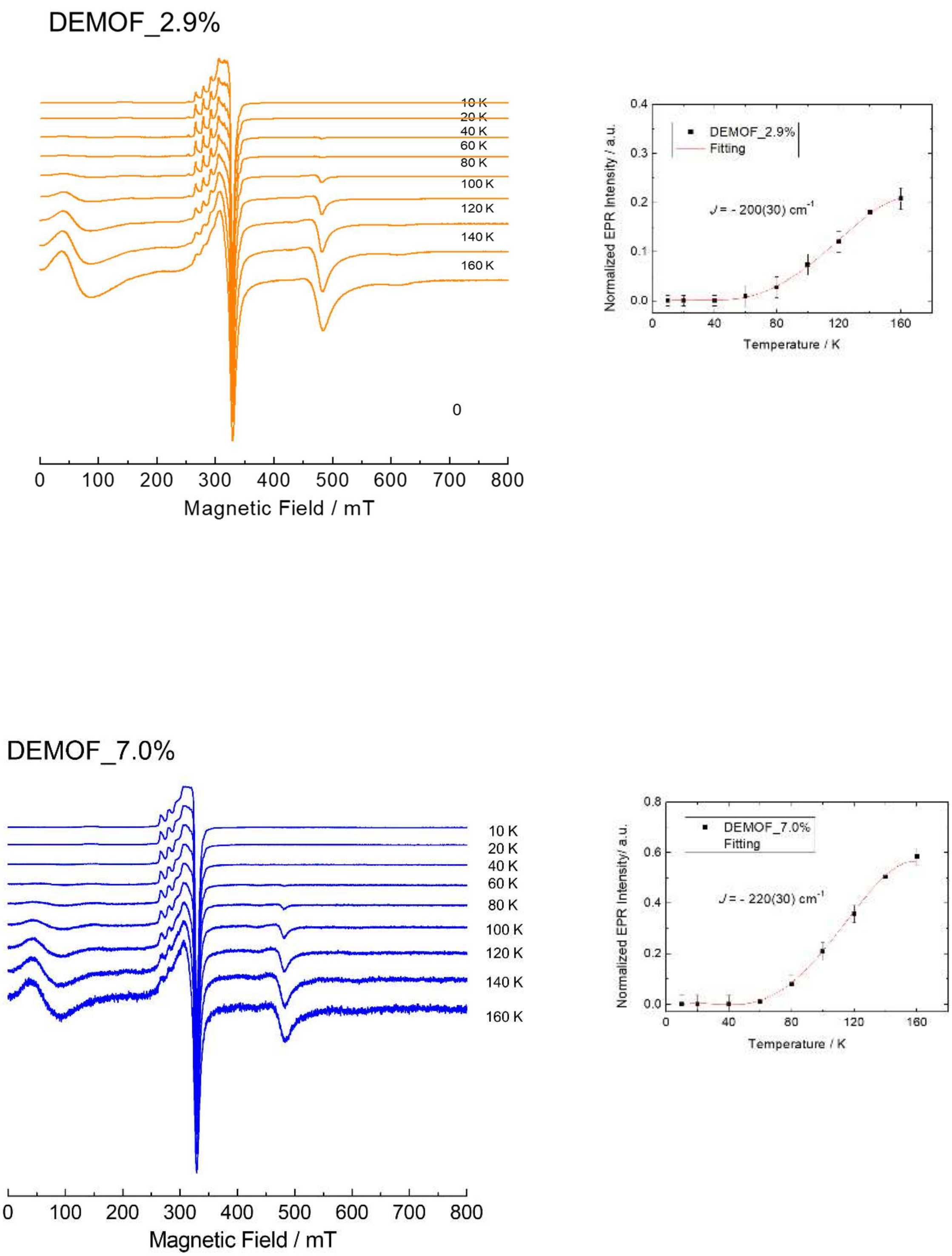

Figure S18. Temperature dependence of X-band *cw* EPR spectra for ReMOF and DEMOF samples. All fitted data of these samples according to Bleaney-Bowers equation is also indicated.

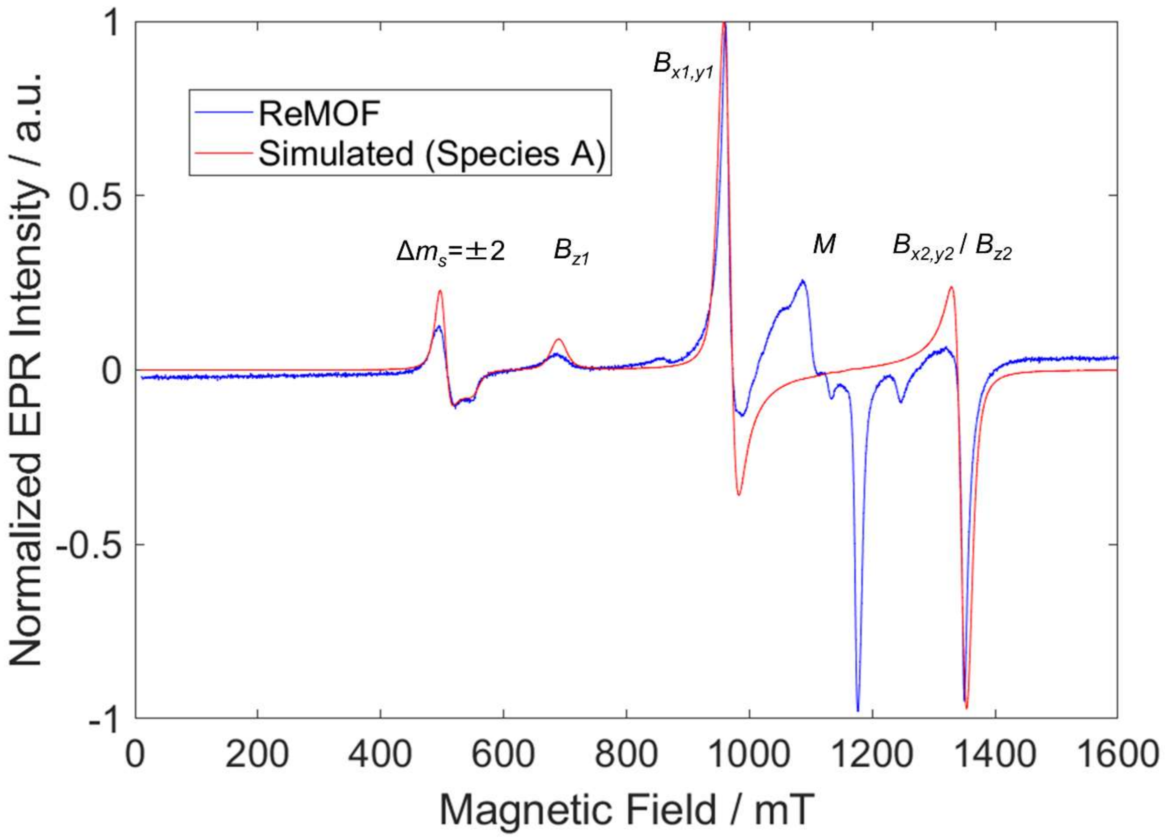

Figure S19. Q-band *cw* EPR spectra for ReMOF sample recorded at 110 K. The red line represents the simulated spectrum for species A. *M* indicates the signal of a mononuclear Cu^{II} species.

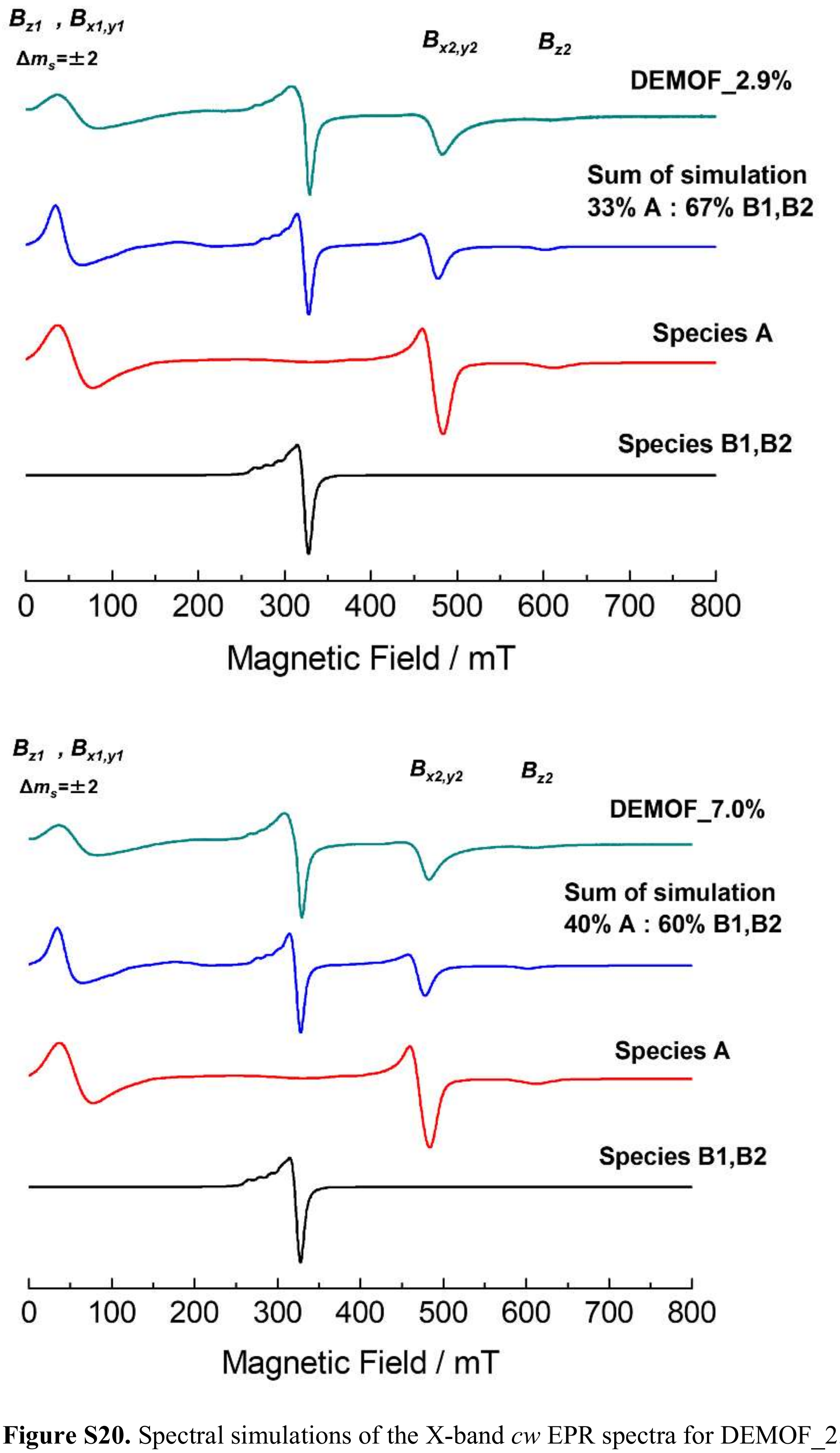

Figure S20. Spectral simulations of the X-band *cw* EPR spectra for DEMOF_2.9% (top) and DEMOF_7.0% samples (bottom) recorded at 160 K.

S13. Gas Adsorption, Isosteric Heat of adsorption

High resolution low-pressure isotherms up to 1 bar were measured at 24 K and 67 K to 87 K using a Belsorp max (Microtrac MRB) equipped with a closed cycle helium cryostat. Before the measurement the sample (50-100 mg) was activated overnight in dynamic vacuum. The measurement was conducted in the pressure range $p/p_0 = 10^{-5} - 1$. Data evaluation was performed using BEL Master 6.3.0.0 software. Helium gas of 99.999% purity was used for determination of the dead volume after each measurement and H_2, CO_2 gas of high purity 99.999% was used for physisorption.

Pore Size Distribution: The pore size distribution was calculated based on CO_2 adsorption isotherms at 298 K, p max = 1 bar using a Belsorp max G (Microtrac, Version: 1.1.0) equipped with a thermostat. BELmaster version 7.3.2.0 software with computer simulation method (HK: Horvath and Kawazoe method)[21] was utilized for calculating the pore size distribution. CO_2 gas of high purity 99.999% used for physisorption. Parameter used Interpolated Curve (3-dimensional Spline Curve), Adsorbate liquid density: 0.7130 g cm-3, Analysis Parameter utilized from BELmaster version 7.3.2.0 software: CO2-C(298K)-BEL.HKS.

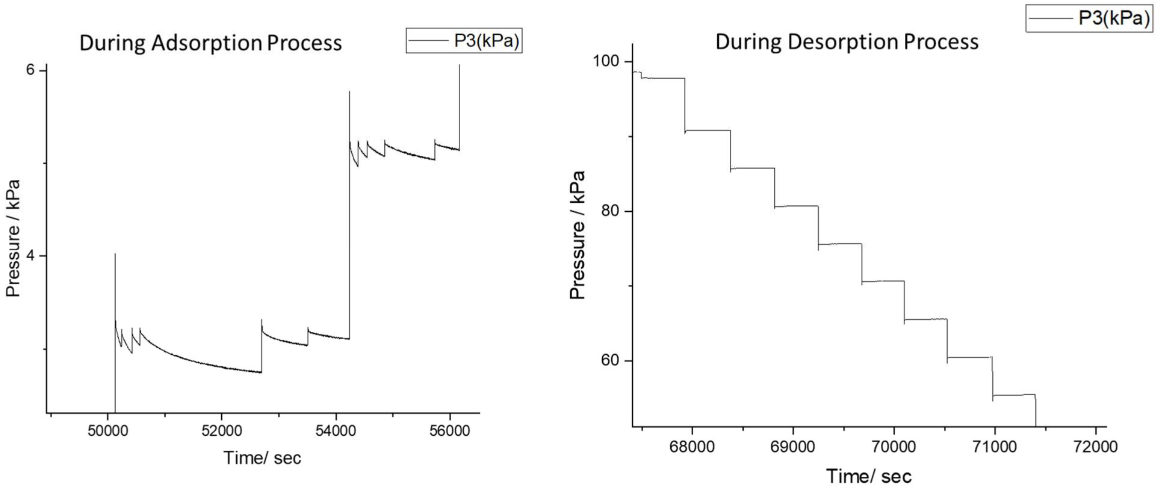

Figure S21. Pressure vs. time plot during adsorption and desorption processes of DEMOF_7.0%.

Figure S21 demonstrates that, while the desorption process is quick, adsorption needs a long time (about 1 h) for equilibration. Thus, desorption isotherms were employed in the context of this study for additional analysis.

Determination of the heat of desorption ΔH_{des} ($= -\Delta H_{ads}$) was carried out using Clausius-Clapeyron approach[18]. ReMOF and DEMOF samples exhibit Langmuir-Freundlich-type isotherms under the aforementioned conditions, which can be fitted using the Langmuir-Freundlich equation (1),

$$n = \frac{a \, . \, b \, . \, p^c}{1+b . \; p^c} \quad (1)$$

where n is the amount of adsorbed material (loading) in mmol g^{-1}, p denotes the pressure in kPa, a denotes the maximum loading in mmol g^{-1}, b denotes the affinity constant ($1/kPa^c$), and c denotes the heterogeneity exponent, the multiplication of bp^c should be dimensionless. We have calculated the respective pressures for respective temperatures T consecutively at the same loading n by plugging in the respective values for a, b and c derived from the Langmuir-Freundlich fit, then compared the pressures at isosteric conditions, that is at the same uptake of the adsorbate. The fitting routine leads to a continuous sequence of loading n vs pressure p data pairs. Following the fitting process, the isotherms for n vs p data sets with the same loading n are evaluated at each temperature. This method is known as "isosteric" approach, which relies on the Clausius-Clapeyron equation (2) given below

$$\Delta H_{ads}(n) = -R\ \ln(\frac{P_2}{P_1})\frac{T_1.T_2}{(T_2-T_1)} \quad (2)$$

with R = ideal gas constant (R = 8.314 J mol^{-1} K^{-1}).

Plotting ln (p) against 1/T for the isosteric adsorptions, that is, given equal n at the three temperatures, yields the isosteric enthalpy of adsorption using the aforementioned equation (2) (see Figures S26-S28)

$$\Delta H_{ads}(n) = R(\frac{\partial \ln(P)}{\partial \frac{1}{T}}) \quad (3)$$

As a result, for various uptakes, ln(p) is plotted against the reciprocal temperature 1/T. The slope of the linear fit to this data for each uptake is proportional to the isosteric heat of adsorption.

Complete gas adsorption-desorption isotherms of ReMOF and DEMOFs at different temperatures

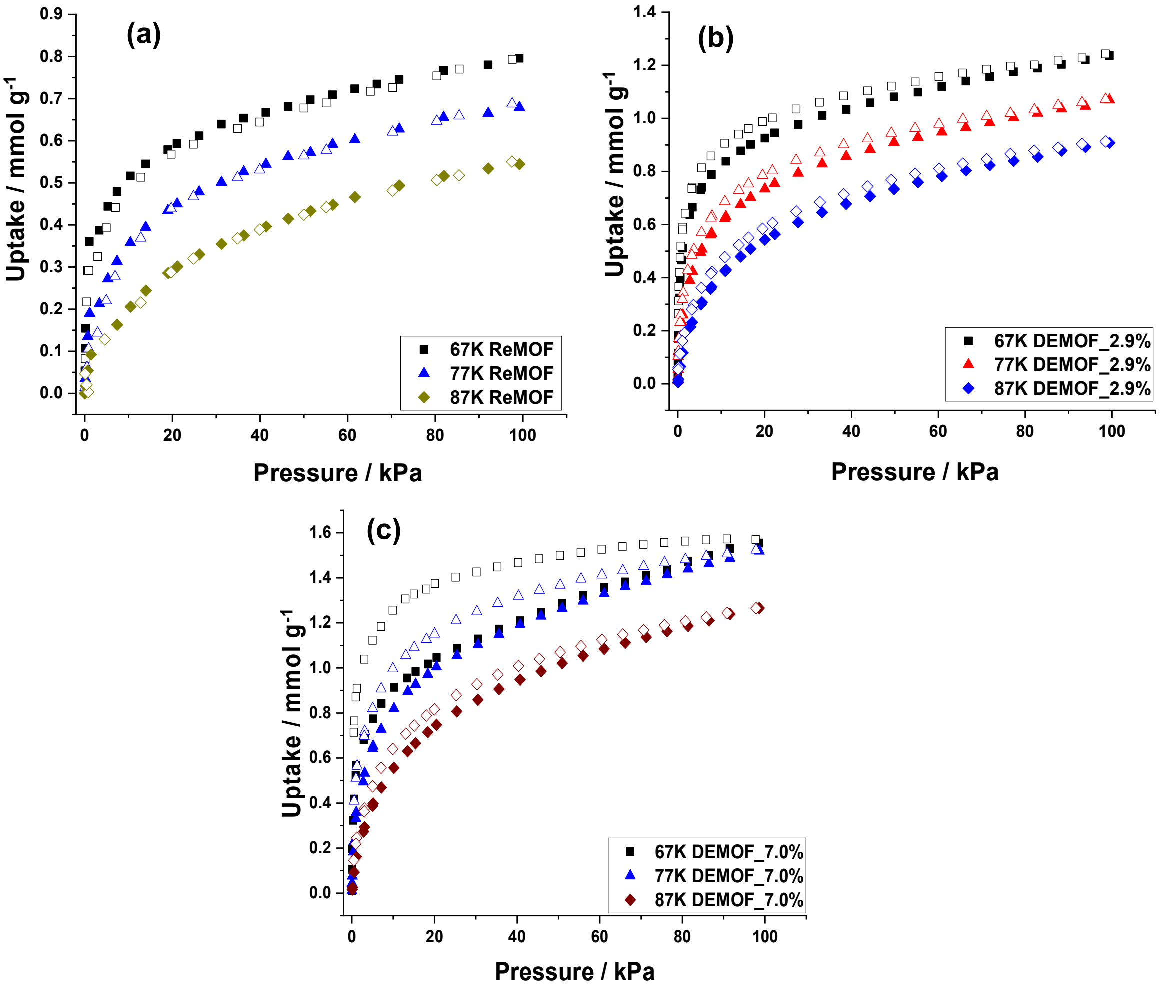

Figure S22. H_2 Adsorption-desorption isotherms of (a) ReMOF, (b) DEMOF_2.9% and (c) DEMOF_7.0% recorded at 67 K, 77 K and 87 K. Filled symbols - adsorption; empty symbols - desorption.

Fitting of isotherms using Freundlich–Langmuir fit.

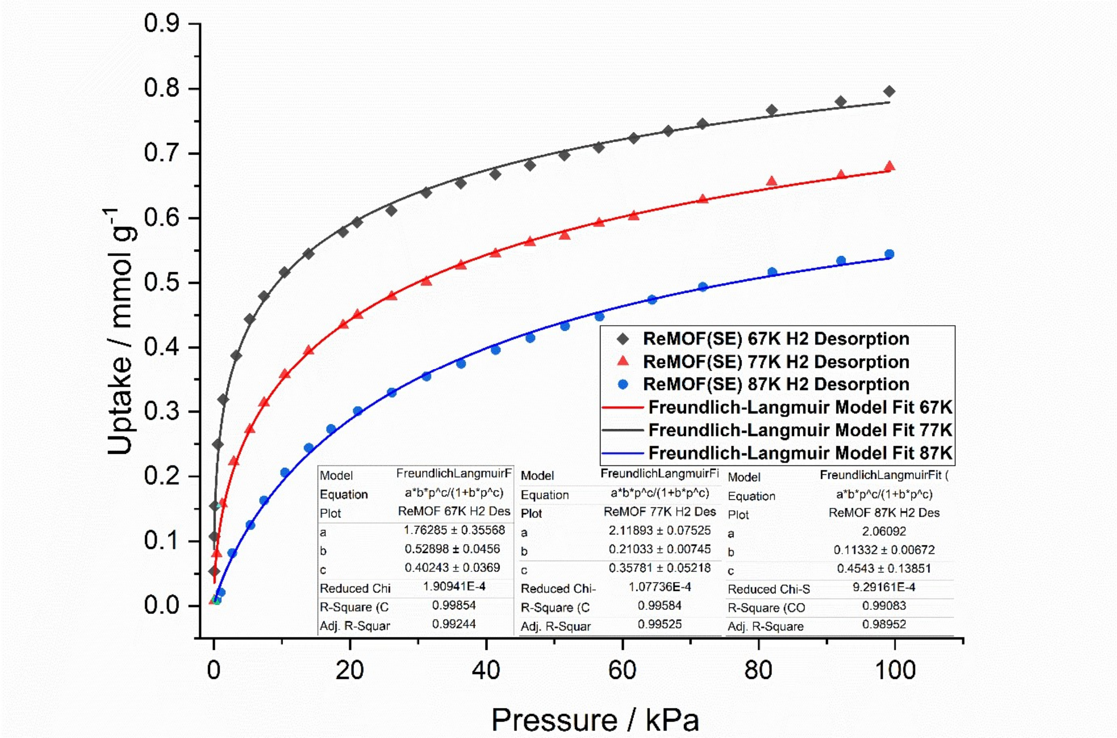

Figure S23. Freundlich-Langmuir fit for H_2 desorption isotherms of ReMOF at 67 K, 77 K and 87 K.

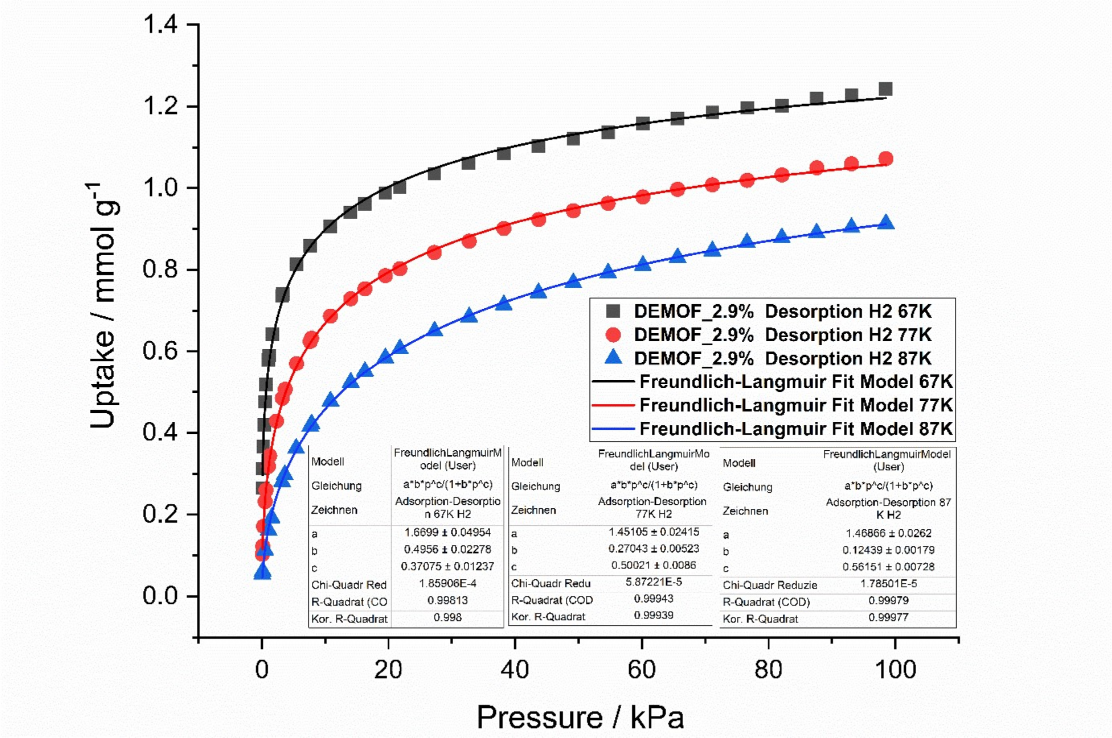

Figure S24. Freundlich-Langmuir fit for H_2 desorption isotherms of DEMOF_2.9% at 67 K, 77 K and 87 K.

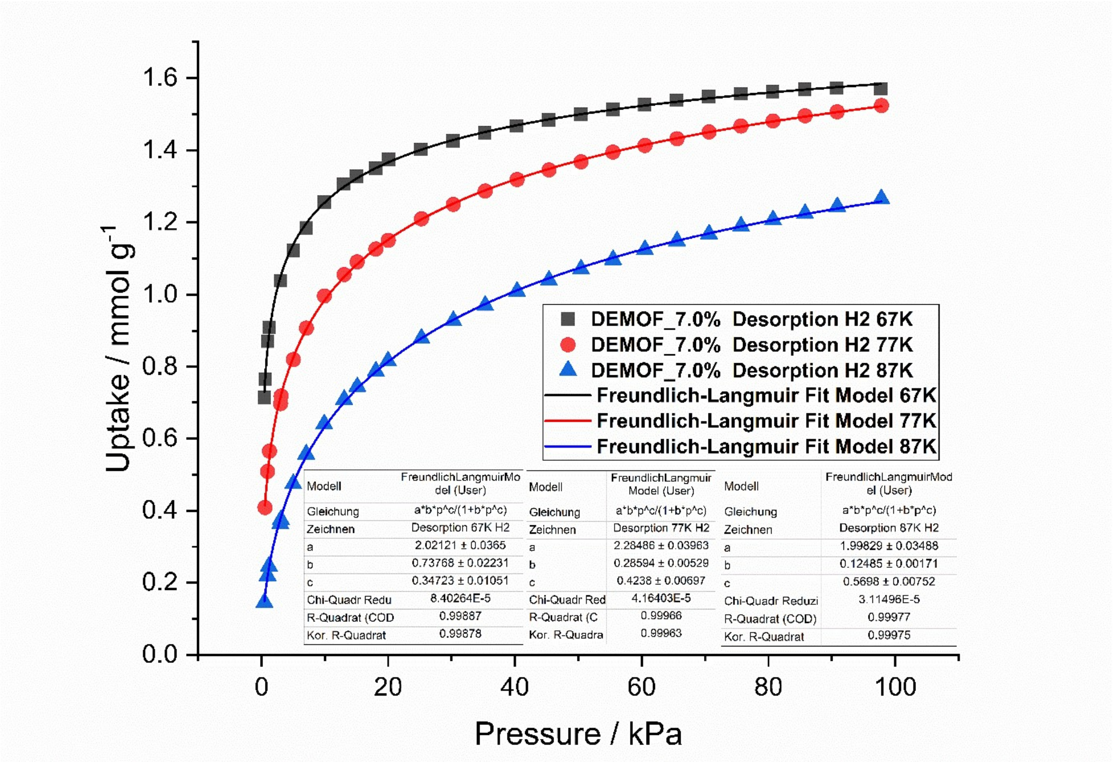

Figure S25. Freundlich-Langmuir fit for H_2 desorption isotherms of DEMOF_7.0% at 67 K, 77 K and 87 K.

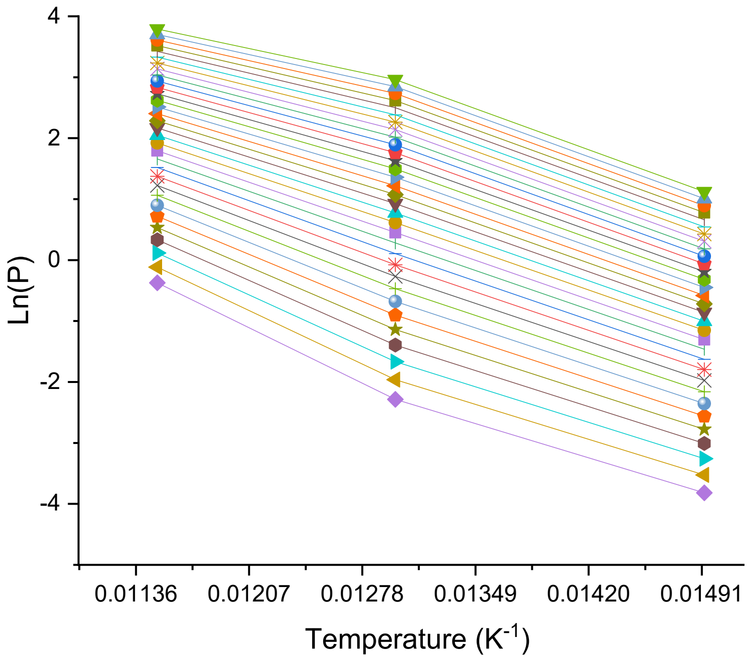

Figure S26. ReMOF isosteric plot of ln (p) against 1/T for different loadings n (in mmol g^{-1}) at 67, 77 and 87 K.

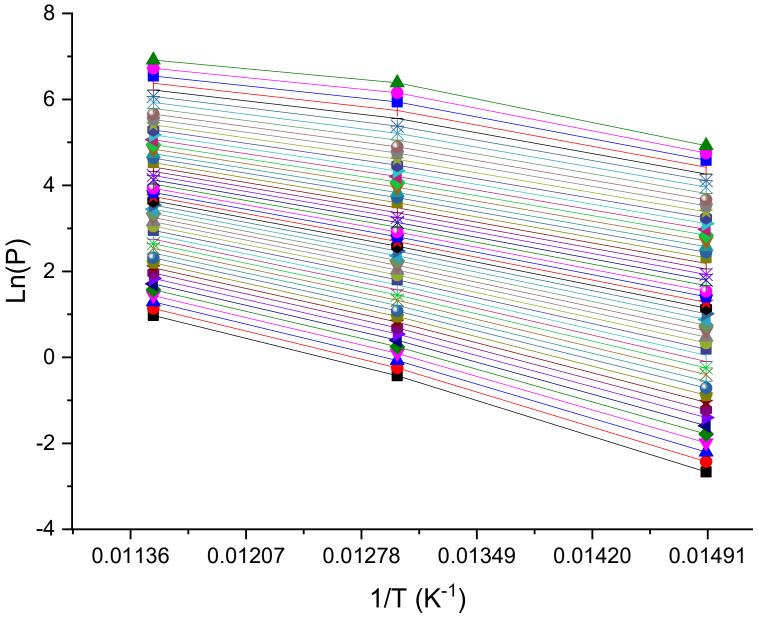

Figure S27. DEMOF_2.9% Isosteric plot of ln (p) against 1/T for different loadings n (in mmol g^{-1}) at 67, 77 and 87 K

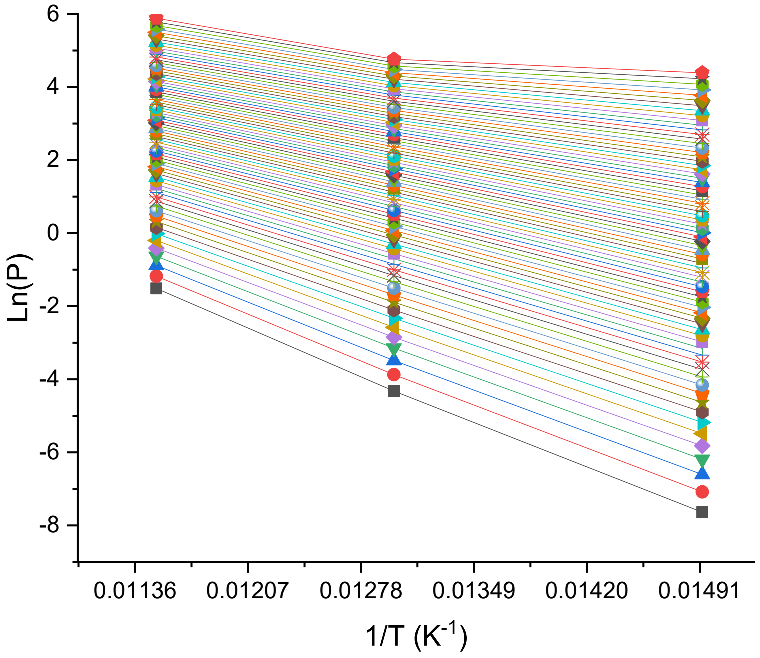

Figure S28. DEMOF_7.0% Isosteric plot of ln (p) against 1/T for different loadings n (in mmol g^{-1}) at 67, 77 and 87K.

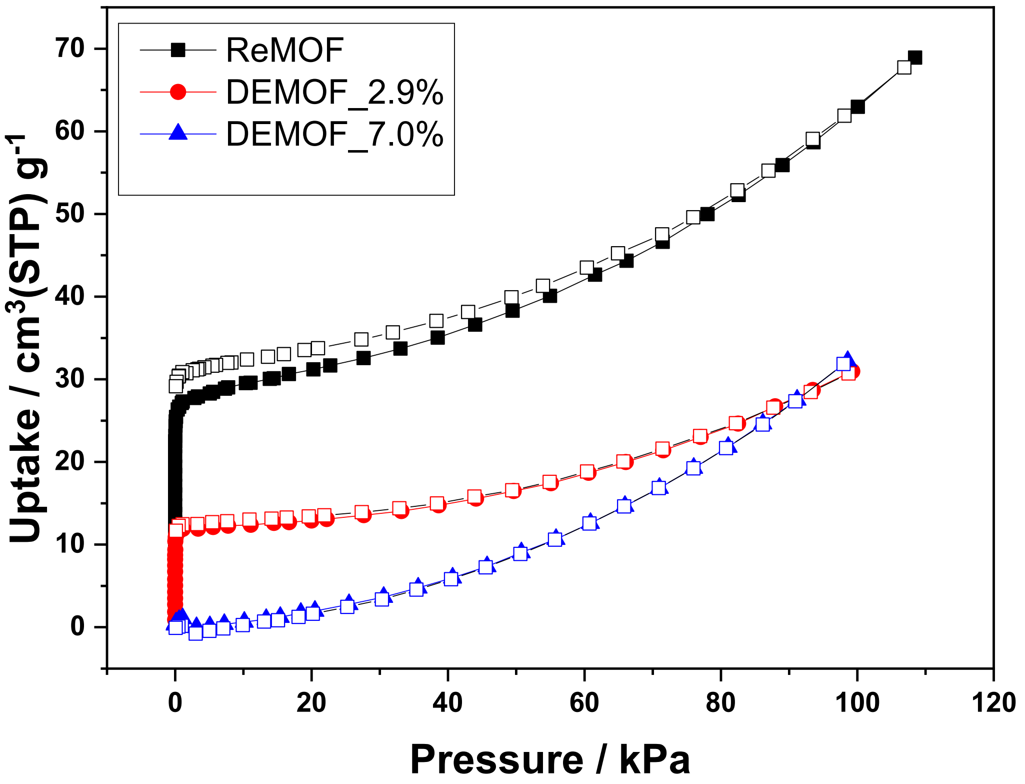

Figure S29. H_2 adsorption-desorption at 24 K of ReMOF, DEMOF_2.9% and DEMOF_7.0%. Filled symbols - adsorption; empty symbols - desorption.

CO_2 Gas adsorption-desorption isotherms and Horvath and Kawazoe (HK) plot for pore size distribution and pore volume

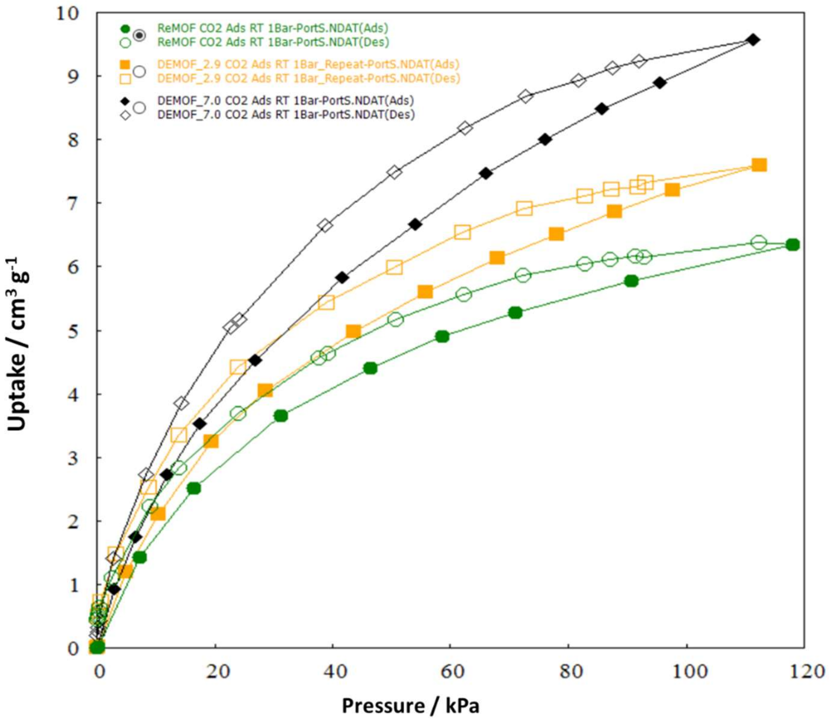

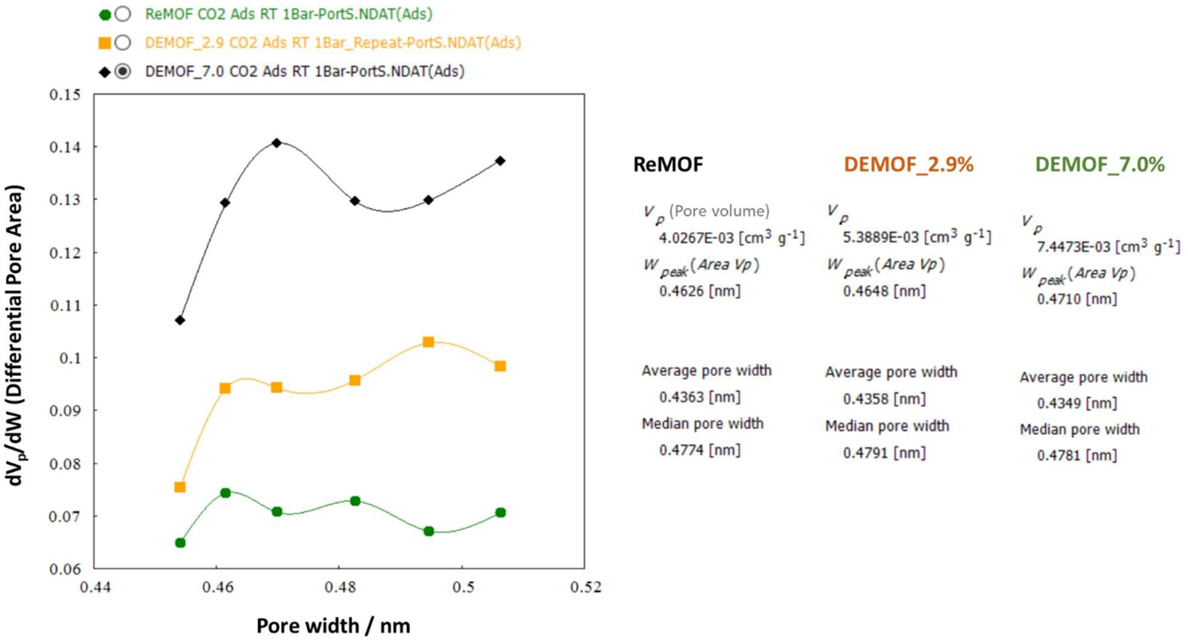

Figure S30. CO_2 adsorption-desorption isotherms at 298 K up to 1 bar (top, filled symbols - adsorption; empty symbols - desorption), pore size distribution and pore volume of ReMOF and DEMOFs samples (bottom) calculated using HK method.

The data depicted in **Figures S22** and **S30** (top) reveal a progressive increase in H_2 and CO_2 gas adsorption capacities transitioning from ReMOF to DEMOF samples. For both cases, CO_2 and H_2, this pattern correlates with an enhancement of gas adsorption due to stronger adsorption site at low pressure and also due to enhanced porosity across the samples at higher pressure due to higher defect concentrations. To delve deeper into the porosity characteristics, Horvath-Kawazoe (HK) analysis[21] using CO_2 gas was employed. This approach is particularly suited for studying ultramicroporous MOFs, which cannot be accurately probed using other adsorbates like N_2 at 77 K due to their larger size. CO_2, being a smaller molecule, serves well for assessing the ultramicroporous nature of these materials. A noticeable hysteresis observed across all samples is indicative of the flexible nature of the MOF structure, a phenomenon previously reported in studies involving high-pressure CO_2 gas adsorption analysis.[2]

In **Figure S30 (bottom)**, several key points emerge regarding pore volume and width: The ReMOF exhibits the least total pore volume, quantified at $4.0 \cdot 10^{-3}$ cm³/g. The DEMOF_2.9% displays a modestly greater pore volume of $5.4 \cdot 10^{-3}$ cm³/g, underscoring that even minimal defect incorporation can appreciably influence porosity. DEMOF_7.0% presents a markedly higher pore volume, $7.4 \cdot 10^{-3}$ cm³/g, denoting an increase in porosity correlating with the rise in defect levels. In contrast to the pore volumes, the average pore width values determined for ReMOF, DEMOF_2.9% and DEMOF_7.0% do not show a significant change.

S14. Scanning Electron Microscopy (SEM) Images

SEM images were captured using a Phenom Pharos G2 Desktop FEG-SEM Tabletop field emission gun scanning electron microscope for high quality imaging.

Acceleration voltages: Default: 15 kV

Detector: Back scattered electron detector (standard)

Scanning Electron Microscopy (SEM) was employed to examine the effect of defect engineering on the morphology of ReMOF and DEMOF samples, respectively, particularly after the evaporation of the solvent. The observed particle size varies between 50 μm and 10 μm in length, 4 μm to 7 μm in breadth. The crystallites exhibit a rough texture and numerous cracks, breaking of bigger crystals into smaller particles in the case of ReMOF and DEMOF_2.9% (**Figures S31-S33**) suggests that the framework structure undergoes significant changes in response to solvent evaporation under vacuum. However, we observed that with increasing defective linker concentration the crystallite sizes increase in length, however, their breadth decreases and also the morphology changes from block (ReMOF) to rod shape (DEMOF_7.0%).

The increase in length (**Figures S31-S33)**, ReMOF (14-6.6 μm), DEMOF_2.9% (34-6.4 μm), DEMOF_7.0% (41- 4.8 μm)) and decrease in breadth of the DEMOFs as the defect percentage increases suggests that defects promote growth in one dimension while inhibiting it in others. This could be due to the defects altering the surface energies of different crystal facets, affecting growth rates, or selectively stabilizing certain growth directions.

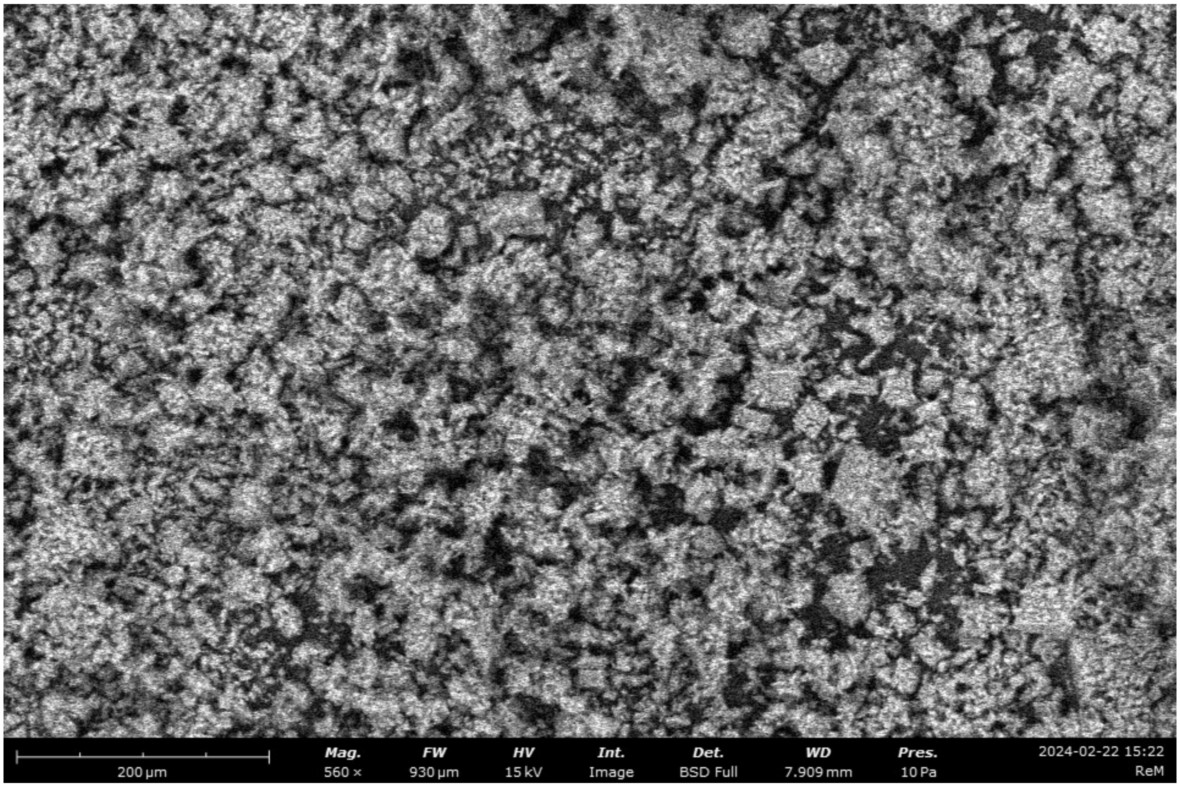

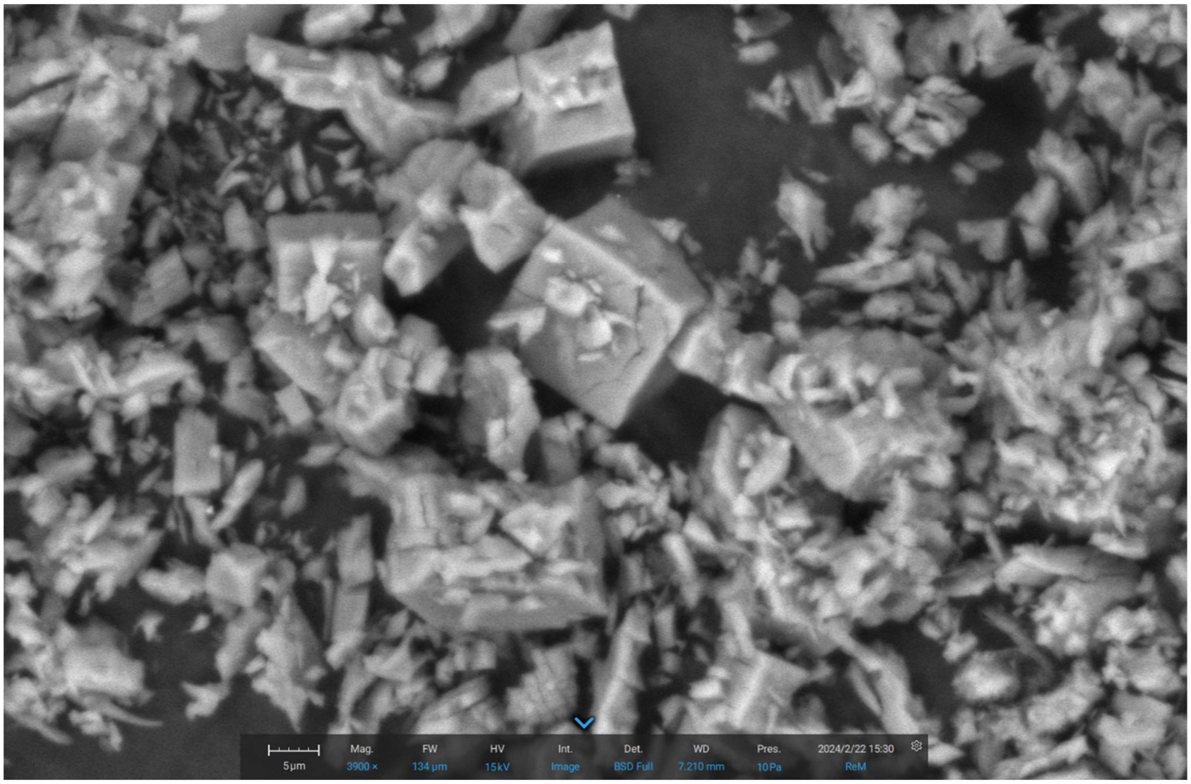
5μm
Mag.
3900 ×
FW
134 μm
HV
15kV
Int.
Image
Det.
BSD Full
WD
7.210 mm
Pres.
10Pa
2024/2/22 15:30
ReM

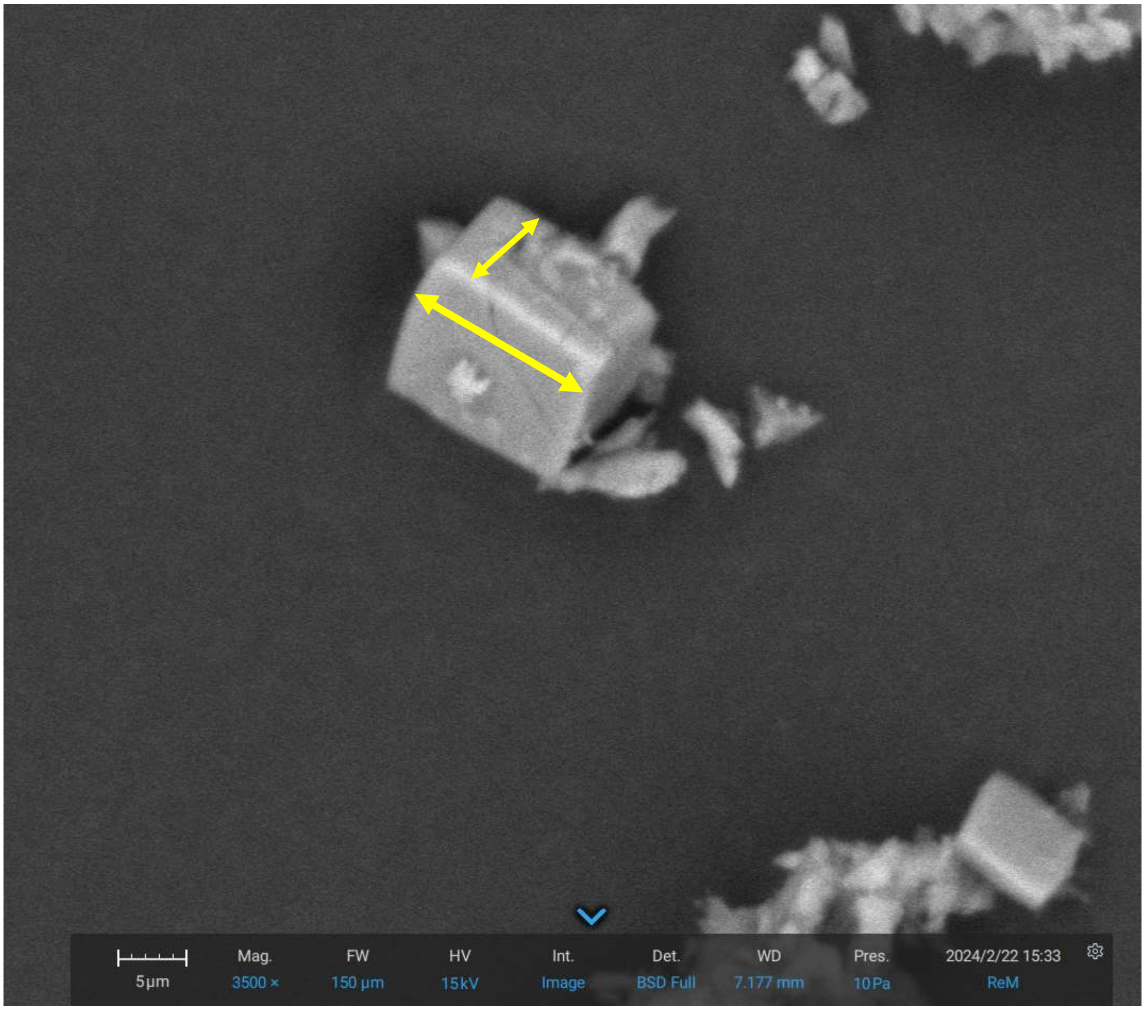

Figure S31. SEM images of ReMOF

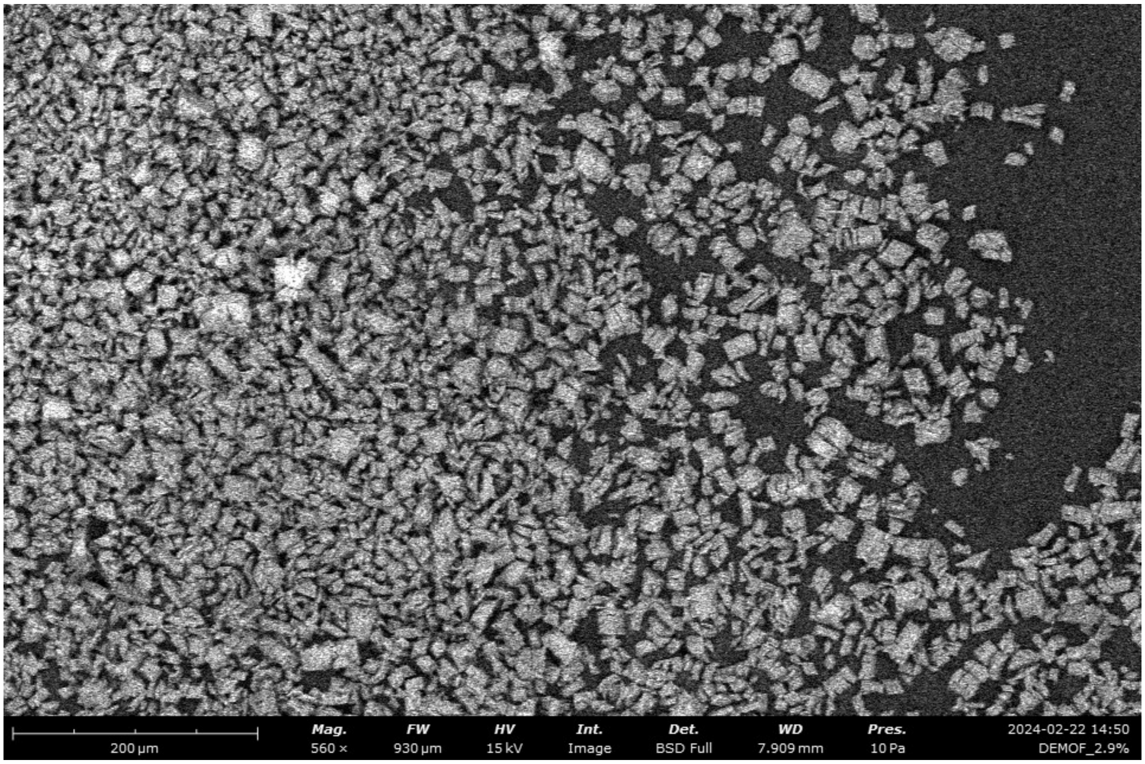
200 µm
Mag. 560 ×
FW 930 µm
HV 15 kV
Int. Image
Det. BSD Full
WD 7.909 mm
Pres. 10 Pa
2024-02-22 14:50
DEMOF_2.9%

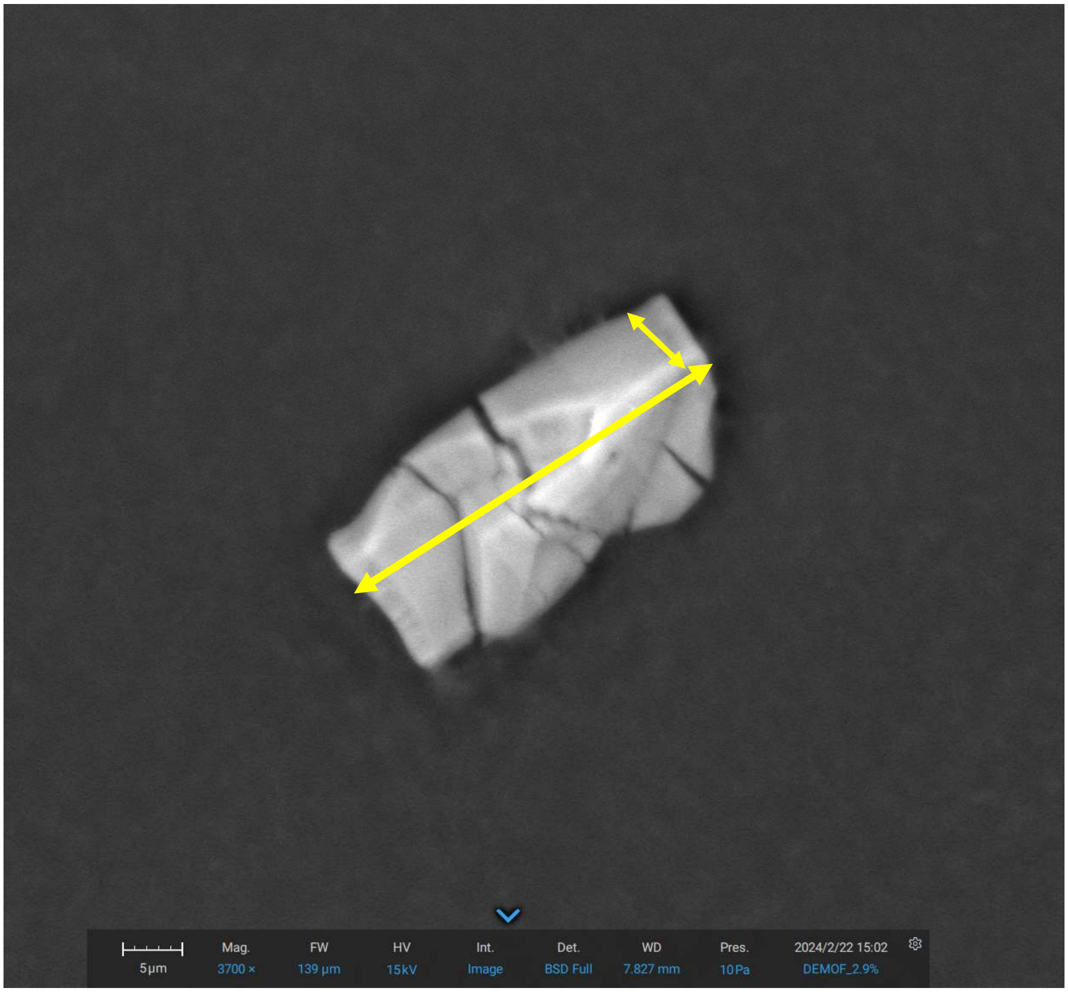

Figure S32. SEM images of DEMOF_2.9%

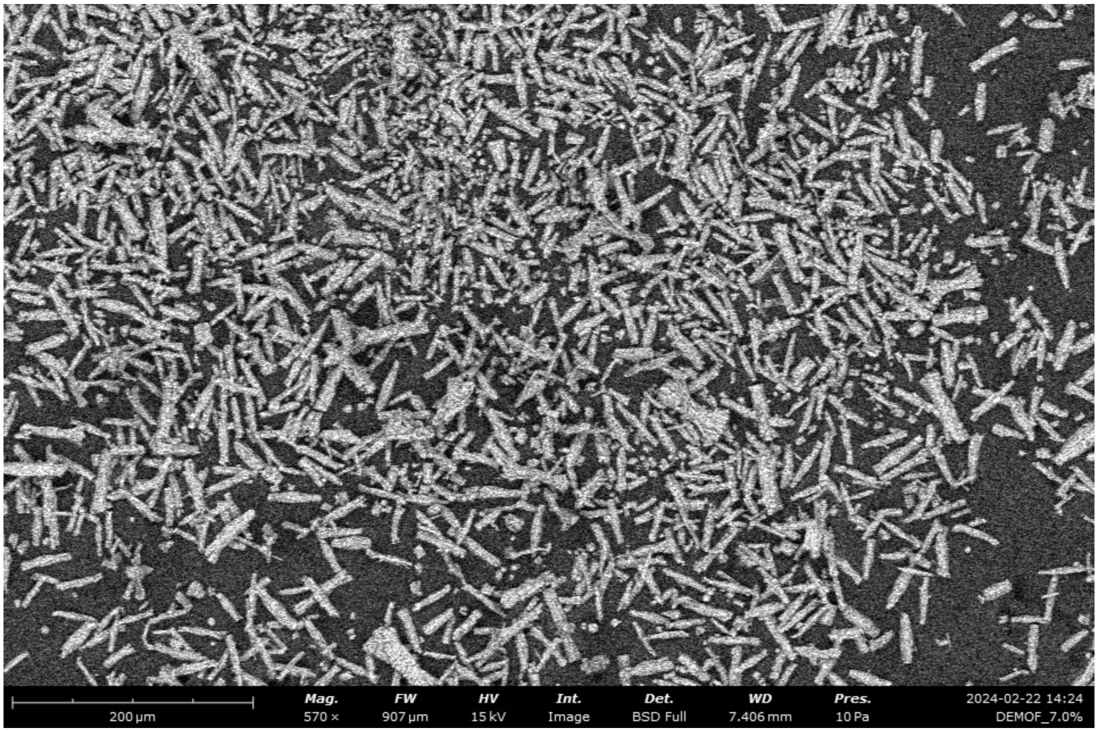
200 μm
Mag. 570 ×
FW 907 μm
HV 15 kV
Int. Image
Det. BSD Full
WD 7.406 mm
Pres. 10 Pa
2024-02-22 14:24
DEMOF_7.0%

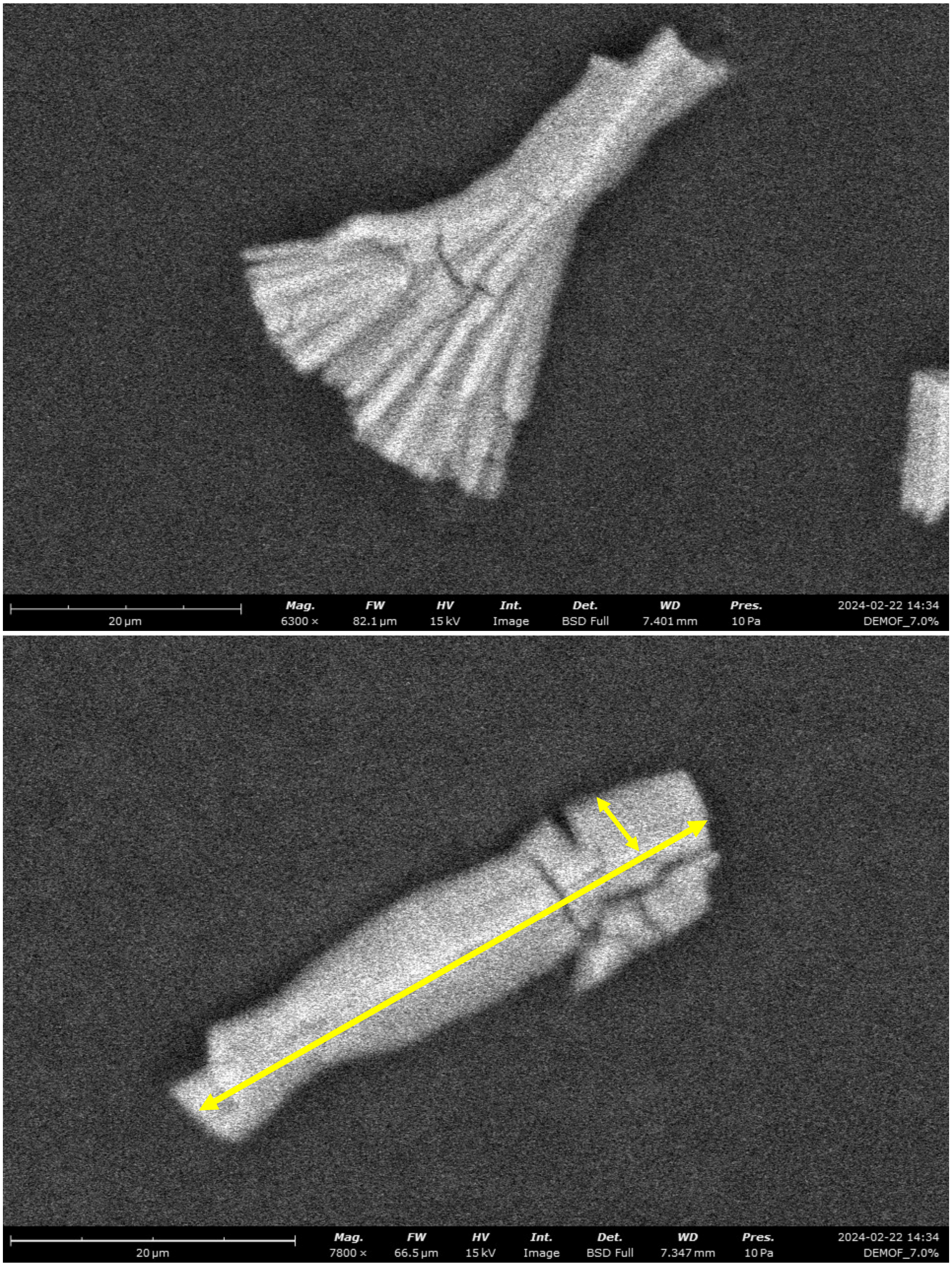

Figure S33. SEM images of DEMOF_7.0%

S15. Elemental Analysis

CHN elemental analyses were carried out on a VARIO EL analyzer (Elementar). The copper content was determined by ICP-OES analyses performed on an Optima 8000 instrument (Perkin Elmer). For sample preparation, the MOFs were dissolved in nitric acid. All the samples were solvent exchanged with MeOH and evacuated before analysis.

Table S5: Elemental analysis results of ReMOF, DEMOF_2.9% and DEMOF_7.0%.

Sample	Formula		Cu (ICP-OES)	C	N	H
ReMOF	$[Cu_2(^1L)_2]$	Calculated	20.6	42.8	13.6	2.3
		Measured	20.1	41.3	13.3	2.3
DEMOF_2.9%	$[Cu_2(^1L_{0.971}{}^2L_{0.029})_2]$	Calculated	20.7	42.9	13.7	2.3
		Measured	20.8	41.2	13.1	2.3
DEMOF_7.0%	$[Cu_2(^1L_{0.93}{}^2L_{0.07})_2]$	Calculated	20.8	42.9	13.7	2.3
		Measured	20.7	40.5	13.0	2.4

S16. References

(1) Lässig, D.; Lincke, J.; Krautscheid, H. Highly functionalised 3,4,5-trisubstituted 1,2,4-triazoles for future use as ligands in coordination polymers. *Tetrahedron Lett.* **2010**, *51*, 653–656.

(2) Kobalz, M. et al. Paddle wheel based triazolyl isophthalate MOFs: Impact of linker modification on crystal structure and gas sorption properties. *Inorg. Chem.* **2016**, *55*, 3030–3039.

(3) Zhao, Y.; Zhang, J. Microstrain and grain-size analysis from diffraction peak width and graphical derivation of high-pressure thermomechanics. *J. Appl. Crystallogr.* **2008**, *41*, 1095–1108.

(4) Bruker AXS. TOPAS Version 5, Karlsruhe, 2014.

(5) Luo, Q. X. et al. PdCl2 immobilized on metal-organic framework CuBTC with the aid of ionic liquids: Enhanced catalytic performance in selective oxidation of cyclohexene. *RSC Adv.* **2016**, *6*, 33048–33054.

(6) Hadjiivanov, K. I. et al. Power of infrared and Raman spectroscopies to characterize metal-organic frameworks and investigate their interaction with guest molecules. *Chem. Rev.* **2021**, *121*, 1286–1424.

(7) Nivetha, R. et al. Cu based metal organic framework (Cu-MOF) for electrocatalytic hydrogen evolution reaction. *Mater. Res. Express* **2020**, 7, 114001.

(8) Pashchevskaya, N. V. et al. Effect of the condition of synthesis on the composition and structure of copper(II) complexes with benzimidazole. *Russ. J. Inorg. Chem.* **2010**, *55*, 1425–1432.

(9) Reyer, A. et al. Ramanspectroscopic investigation of tannin-furanic rigid foams. *Vibrational Spectroscopy* **2016**, *84*, 58–66.

(10) Muniz-Miranda, M. et al. SERS and DFT study of copper surfaces coated with corrosion inhibitor. *Beilstein J. Nanotechnol.* **2014**, *5*, 2489–2497.

(11) Fang, Z. et al. Structural complexity in metal-organic frameworks: Simultaneous modification of open metal sites and hierarchical porosity by systematic doping with defective linkers. *J. Am. Chem. Soc.* **2014**, *136*, 9627–9636.

(12) NETZSCH-Gerätebau GmbH. Proteus Analysis; NETZSCH-Gerätebau GmbH: Selb, Germany, 2009.

(13) Fairley, N. et al. Systematic and collaborative approach to problem solving using X-ray photoelectron spectroscopy. *Appl. Surf. Sci. Adv.* **2021**, *5*, 100112.

(14) St. Petkov, P. et al. Defects in MOFs: A thorough characterization. *ChemPhysChem* **2012**, *13*, 2025–2029.

(15) Wang, J. et al. Defect-engineered metal-organic frameworks: A thorough characterization of active sites using CO as a probe molecule. *J. Phys. Chem. C* **2021**, *125*, 593–601.

(16) Roy, K.; Gopinath, C. S. UV photoelectron spectroscopy at near ambient pressures: Mapping valence band electronic structure changes from Cu to CuO. *Anal. Chem.* **2014**, *86*, 3683–3687.

(17) Stoll, S.; Schweiger, A. EasySpin, a comprehensive software package for spectral simulation and analysis in EPR. *J. Magn. Reson.* **2006**, *178*, 42–55.

(18) Nuhnen, A.; Janiak, C. A practical guide to calculate the isosteric heat/enthalpy of adsorption: Via adsorption isotherms in metal-organic frameworks, MOFs. *Dalton Trans.* **2020**, *49*, 10295–10307.

(19) Hemström, P.; Irgum, K. Hydrophilic interaction chromatography. *J. Sep. Sci.* **2006**, *29*, 1784–1821.

(20) Lu, Y.; Zhang, G.; Zhou, H.; Cao, S.; Zhang, Y.; Wang, S.; Pang, H. Enhanced Active Sites and Stability in Nano-MOFs for Electrochemical Energy Storage through Dual Regulation by Tannic Acid. *Angew. Chem.Int. Ed*. 2023, *62*, e2023110

(21) Horváth. G, Kawazoe. K, Method for the calculation of effective pore size distribution in molecular sieve. *Carbon. J. Chem. Eng. Japan* **1983**, *16*, 470.
